# The effect of telephone health coaching and remote exercise monitoring for peripheral artery disease (TeGeCoach) on health care cost and utilization: results of a randomized controlled trial

**DOI:** 10.1007/s10198-023-01616-4

**Published:** 2023-07-10

**Authors:** Dirk Heider, Farhad Rezvani, Herbert Matschinger, Jörg Dirmaier, Martin Härter, Lutz Herbarth, Patrick Steinisch, Hannes Böbinger, Franziska Schuhmann, Gundula Krack, Thomas Korth, Lara Thomsen, Daniela Patricia Chase, Robert Schreiber, Mark-Dominik Alscher, Benjamin Finger, Hans-Helmut König

**Affiliations:** 1 Department of Health Economics and Health Services Research, University Medical Center Hamburg-Eppendorf, Martinistrasse 52, 20246 Hamburg, Germany; 2 Department of Medical Psychology, University Medical Center Hamburg-Eppendorf, Hamburg, Germany; 3 KKH Kaufmännische Krankenkasse Statutory Health Insurance, Hannover, Germany; 4 TK Techniker Krankenkasse Statutory Health Insurance, Hamburg, Germany; 5 mhplus Krankenkasse Statutory Health Insurance, Ludwigsburg, Germany; 6 IEM GmbH, Aachen, Germany; 7 Philips GmbH, Hamburg, Germany; 8 Robert Bosch Gesellschaft Für Medizinische Forschung mbH, Bosch-Institute of Clinical Pharmacology, Dr. Margarete Fischer, Stuttgart, Germany

**Keywords:** Peripheral artery disease, Home-based exercise program, Randomised controlled trial, Intention-to-treat, Health care use and costs, I

## Abstract

**Background:**

Peripheral artery disease (PAD) is the third most prevalent atherosclerotic cardiovascular disease. In 2016, costs per patient associated with PAD exceeded even the health-economic burden of coronary heart disease. Although affecting over 200 million people worldwide, a clear consensus on the most beneficial components to be included in home-based exercise programs for patients with peripheral artery disease is lacking. The aim of the study was to examine the health care use and costs caused by the 12-month patient-centered ‘Telephone Health Coaching and Remote Exercise Monitoring for Peripheral Artery Disease’ (TeGeCoach) program in a randomized controlled trial.

**Methods:**

This is a two-arm, parallel-group, open-label, pragmatic, randomized, controlled clinical trial (TeGeCoach) at three German statutory health insurance funds with follow-up assessments after 12 and 24-months. Study outcomes were medication use (daily defined doses), days in hospital, sick pay days and health care costs, from the health insurers’ perspective. Claims data from the participating health insurers were used for analyses. The main analytic approach was an intention-to-treat (ITT) analysis. Other approaches (modified ITT, per protocol, and as treated) were executed additionally as sensitivity analysis. Random-effects regression models were calculated to determine difference-in-difference (DD) estimators for the first- and the second year of follow-up. Additionally, existing differences at baseline between both groups were treated with entropy balancing to check for the stability of the calculated estimators.

**Results:**

One thousand six hundred eighty-five patients (Intervention group (IG) = 806; Control group (CG) = 879) were finally included in ITT analyses. The analyses showed non-significant effects of the intervention on savings (first year: − 352€; second year: − 215€). Sensitivity analyses confirmed primary results and showed even larger savings.

**Conclusion:**

Based on health insurance claims data, a significant reduction due to the home-based TeGeCoach program could not be found for health care use and costs in patients with PAD. Nevertheless, in all sensitivity analysis a tendency became apparent for a non-significant cost reducing effect.

**Trial registration:**

NCT03496948 (www.clinicaltrials.gov), initial release on 23 March 2018

**Supplementary Information:**

The online version contains supplementary material available at 10.1007/s10198-023-01616-4.

## Introduction

Becoming one of the leading causes of disability and death [1, 2], peripheral Artery Disease (PAD) is the third most prevalent atherosclerotic cardiovascular disease, affecting over 200 million people worldwide. With estimates of 5.4% and 18.6% of individuals aged from 45 to 49 and 85–89 years being affected, PAD is clearly more prevalent in the elderly population [1, 2].

With a sharp increase by nearly 25% between 2000 and 2010, the amount of people with PAD in the general population has risen rapidly in recent years [2]. Between 2005 and 2009 the amount of PAD-related hospitalizations increased by 20.7% in the German population, while hospital reimbursement costs for the treatment of PAD increased by an amount of 21% from €2.1 billion to €2.6 billion from 2007 to 2009 [3]. Relevant risk factors for PAD are tobacco smoking and diabetes, followed by high cholesterol, hypertension, history of cardiovascular disease (i.e., coronary heart disease, stroke) and chronic kidney disorder [1, 2, 4, 5]. Exceeding even the health-economic burden of coronary heart disease, the costs for PAD were €5,552 per patient in 2016, while those for coronary heart disease were amounted to €4,008. When both diseases were present [6], annual costs of even €8,067 per patient were reached. Revascularizations cause about half of the PAD associated hospital costs [7]. Thereby the severity of the PAD is associated with rising costs. In Fontaine stages 3 and 4, health care for PAD resulted in higher annual costs in the U.S with a total of $3.5 billion as opposed to $2.8 billion for patients in Fontaine stages 1 and 2 [8]. Being attributed to higher rates of presenteeism and absenteeism, indirect mortality and morbidity-related costs have to be taken into account additionally to the direct costs arising from the medical care of PAD [9]. Nevertheless, indirect costs may be considered rather low, since the average age of PAD patients is relatively high, and, therefore, many of them are no longer active in the labor market [10].

The ageing of the population and a further increasing prevalence of risk factors for PAD likely leads to an aggravation of financial and organizational barriers in the future. Therefore, the actual implementation of supervised exercise programs (SEPs) in usual care will be confronted with more difficulties, while the economic burden on healthcare systems caused by PAD might be foreseeable on a high and likely growing level.

Partly ineffective and insufficient usual care of PAD has resulted in a poorly served patient population and high mortality rates which encouraged the emergence of structured home-based exercise programs (HEPs) in case SEPs are not available or impractical to deliver [11]. As HEPs have been found to improve walking impairment [12], they are recommended as second-line therapy [13], supported by a high level of evidence in clinical practice guidelines. Although various studies have shown that HEPs may improve quality of life, walking ability and claudication symptoms [14–18], HEPs are still considered inferior to SEPs [19–21], while unstructured programs with a solely general walking advice have been shown to be ineffective [22]. Nevertheless, some other studies found no effects or only mixed results of structured HEPs [23–26]. Different study designs, such as different control conditions (e.g., care-as-usual (CAU), attention control), outcomes (e.g., performance-based, patient-reported) and inclusion criteria as well as varying intervention components and risks of bias (e.g., participation bias, attrition bias) may be the reason for inconsistent results in existing studies.

Since there is yet no clear consensus on the most beneficial components of HEPs and for their effects on health care use and costs for PAD patients in general, the TeGeCoach-study examined a 12-month long HEP incorporating motivational interview-based telephone health coaching, attending regular medical check-ups and telemonitoring-guided walking exercise using wearable activity trackers, which differs from other HEPs mostly by a longer duration and a higher intensity of support contact. The aim of the present analysis was to explore the health care use and costs associated with the TeGeCoach intervention for patients with PAD in the real-world management of PAD.

## Methods

### Trial design

**Fig. 1:**
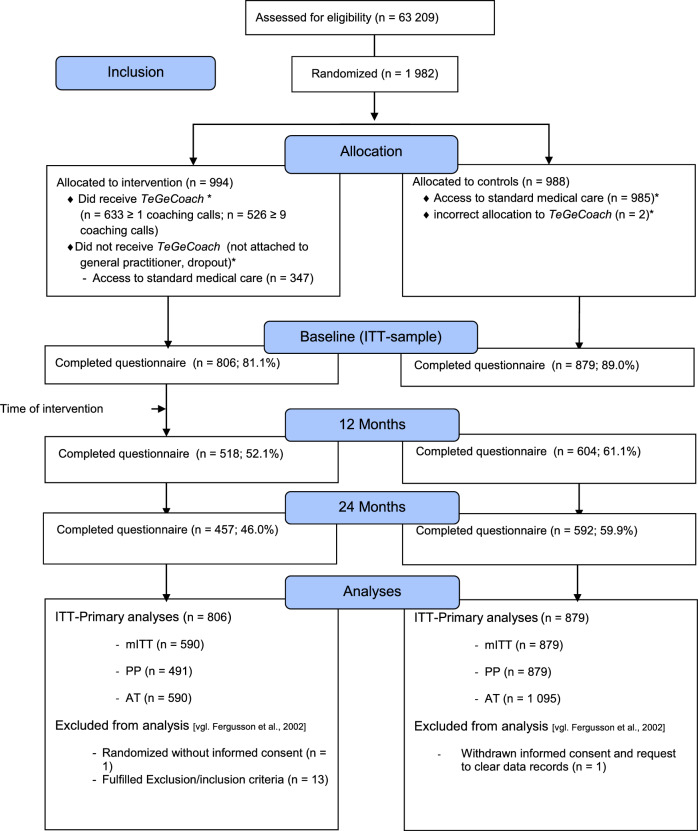
Consort-Flow-Chart ITT

TeGeCoach is a two-arm, parallel-group, open-label, pragmatic, randomized, controlled clinical trial embedded within three German statutory health insurances (KKH Kaufmännische Krankenkasse*,* TK Techniker Krankenkasse, and mhplus Krankenkasse). It was designed to compare the effects of TeGeCoach (intervention arm) to the usual care of PAD (care-as-usual, CAU), conducted in a health insurance system-based setting (Fig. 1). Trial initiation was in 04/2018 when enrolment began and it ended in 02/2021. The recruitment period was 9 months (04/2018—12/2018). TeGeCoach has been registered at www.clinicaltrials.gov (NCT03496948); protocol modifications were added to the trial registry. Ethical approval has been obtained from the ethics committee of the Medical Association Hamburg (Ärztekammer Hamburg). The study was conducted in full compliance with Good Clinical Practice quality standards and in accordance with the Declaration of Helsinki of 2008.

The study protocol was reported in accordance with the CONsolidated Standards Of Reporting Trials (CONSORT) statement [27]; the Standard Protocol Items: Recommendations for Interventional Trials (SPIRIT) statement [28]; the SPIRIT Patient-Reported Outcome (PRO) extension [29]; and the Template for Intervention Description and Replication (TIDieR) checklist [30].

### Study participants

Participants had to meet the following criteria: insured at one of the participating statutory health insurances (KKH Kaufmännische Krankenkasse*,* TK Techniker Krankenkasse, mhplus Krankenkasse); aged between 35 and 80; German-speaking; access to a telephone (landline or mobile); and a primary or secondary diagnosis of PAD at Fontaine stage IIa or IIb within the past 36 months. To increase diagnostic accuracy, participants should have no primary or secondary diagnosis of PAD at Fontaine stage I (asymptomatic) within the past 12 months, and no diagnosis of Fontaine stage III (ischemic rest pain) or IV (ulcer, gangrene) within the last 36 months.

Exclusion criteria for participants were as follows: immobility that goes beyond claudication (Fontaine stage III or IV; inability to carry out intervention); (chronic) physical conditions that interfere with the intervention (e.g., COPD); cognitive disorders (inability to carry out intervention); severe and persistent mental disorders (adherence reasons); suicidality (safety reasons); life-threatening illnesses (safety reasons); active or recent participation in any other PAD intervention trial; ongoing hospitalization; (self-reported) alcoholism and/or other drug dependency (adherence reasons); and heart failure graded NYHA class III and IV (inability to carry out intervention and competing risks).

### Recruitment

Recruitment of participants was managed by the following three German statutory health insurances: KKH Kaufmännische Krankenkasse*,* TK Techniker Krankenkasse and mhplus Krankenkasse. Eligible participants were retrospectively identified through the screening of health insurance claims data, which are routinely collected for reimbursement purposes by statutory health insurances in Germany. Eligible patients were identified using ICD-10 codes from inpatient and outpatient encounters. Given the high number of diagnostic errors and poor coding habits in outpatient settings, exclusion criteria were being checked using inpatient diagnosis codes only.

Eligible participants were contacted by their health insurance company to explain the purpose of the study and to confirm that all criteria for study participation were met by undergoing further screening. Eligible participants received a study information letter that was supplemented with consent and permission forms (i.e. authorization for release of medical reports by the treating physician to the health coach). If interested to participate, they were asked to participate in the study by signing the informed consent and all permission forms, and send them back to their health insurance. Non-responders and insured individuals that were still interested in the study but had not given written consent were being followed up by phone to be reminded of the trial. Once the written consent had been received, a query was submitted to the data warehouse of the respective health insurance which automatically assigned a pseudonym to the participant. No participant was enrolled without full, written informed consent.

All TeGeCoach participants attended regular medical check-ups; participants could elect their preferred physician prior to program start, or were alternatively referred to a physician by their health coach. To encourage physicians to participate, they were entered into an integrated care contract with the respective health insurance that provided financial incentives for the delivery of special medical services throughout the intervention. The enrolment and reimbursement of physicians was coordinated by medicalnetworks (Kassel, Germany), a company that is specialized on the management of integrated care programs (ICPs) within the § 140a volume V of the German Social Security Code (SGB V). If the physician of choice refused to participate, the participant was referred to a nearby physician that had entered into the integrated care contract. Once enrolled, the health coach contacted the physician to discuss their tasks during the course of the study.

### Treatment allocation and blinding

Participants were allocated in a 1:1 ratio to either the TeGeCoach or CAU group, stratified by telemedicine center using a permuted block method within each stratum. In order to prevent selection bias and to eliminate any predictability (allocation concealment), participants were randomly allocated via Sealed Envelope (London, United Kingdom), a secure internet-based randomization service including concealment, stratification and blocking for each health coaching site.

Blinding of care providers (health coaches and treating physicians) and trial participants was not possible because of obvious differences between the intervention and care as usual. However, as supported by the CONSORT guidelines, blinding of the analysis was achieved by withholding information about how the groups were coded and by engaging an independent data analyst.^41^

### Intervention

TeGeCoach is a 12-month patient-centered HEP. It was designed to inspire healthy habits and to change unhealthy habits to improve health outcomes in patients with PAD. Participants were continuously wearing an activity tracker device (KKH and mhplus: AS 95 Pulse by Beurer; TK: Mi Band 2 by Xiaomi) enabling them to self-monitor their physical activity (i.e. number of steps). The data were being automatically transferred to the telemedicine centers so participants could retrieve feedback from their coaches. Participants were assigned to one of three walking plans depending on their functional status and current exercise capacity at baseline: involving either 15, 15–30 or 60 min of walking per day. In order to increase walking speed and distance progressively, participants were asked to walk to maximal tolerable pain and with rests in between, according to the principles of interval training. In order to improve health literacy, patient-tailored topics of interest that are relevant to the management of PAD were being discussed along with the walking exercise.

Therefore, participants were being set up a maximum of nine structured 30–60 min phone calls with their health coach to discuss the progress towards exercise goals, review the wearable activity monitor data and to check their adherence over the course of a 12-month follow-up. At signs of a poor adherence, additional phone calls by the health coaches were warranted to give behavioural support. Also, accompanying supportive informative handouts were given to the participants. An additional 12 months of unstructured follow-up, allowing participants to keep using their wearable activity tracker, followed after the completion of the telephone health coaching.

### Care as usual (CAU)

Patients allocated to CAU received usual medical care from their own physicians. Additionally, participants received PAD patient information brochures from their statutory health insurance. These leaflets provided information about course offerings of the respective health insurances to encourage regular exercise and to promote lifestyle changes, including SEPs (vascular and cardio exercise), physical therapy, nutritional assistance programs, smoking cessation programs, weight loss programs, as well as patient education programs for obesity and diabetes. Participants allocated to TeGeCoach had regular access to usual care and received the same patient information (i.e., leaflets, brochures).

### Study outcomes

Health service use was assessed by medication use (defined daily dose—DDD), days in hospital and sick pay days. DDDs as an indicator for medication consumption across all medicines were available only for those medications prescribed by physicians in the outpatient sector. Health care costs, from the perspective of health insurance, were measured in terms of costs for outpatient physician services, (general practitioners and specialists), outpatient non-physician services (e.g. physiotherapy), medical supplies, inpatient treatment, sick pay, medication, rehabilitation and prevention training. All costs in these nine categories were summed to obtain a variable that represented total costs. Reported outcomes were assessed at baseline (t0), at 12 (t1) and 24 (t2) months’ follow-up. By covering equal periods of 12 months, a reasonable timely analysis and comparison of the outcomes in both study groups will be ensured.

Claims data are routinely collected for the purpose of billing and contain information on all contacts with the health care system (e.g.,: ICD-10 codes; operations and procedure key code—OPS; medication; sick pay). After pseudonymization by the health insurers the claims data were handed to the analysing institution, the University Medical Center Hamburg-Eppendorf. Since based on health insurance claims data, the present paper is concerned with the analysis of economic outcomes of the TeGeCoach exclusively. 

### Statistical analysis

Analyses were in accordance with the CONSORT guidelines and conducted by intention-to-treat (ITT), modified intention-to-treat (mITT), per protocol (PP) and as treated (AS) approaches. As outlined in the study protocol [31], unbalanced ITT was the main analysis. Patients randomized into the intervention group who did not follow the TeGeCoach-program as intended or discontinued it, or for whom no doctor could be named (i.e. who received standard care) were still included in the analyses as allocated. This was complemented by the three other approaches in order to examine the robustness of the results. Thereby, mITT included all patients in the intervention group who had access and actually participated in the TeGeCoach-program (received at least 1 coaching call). Patients who did not have access to the TeGeCoach-program were excluded from the mITT analysis. The PP-analysis included all patients of the intervention group who had completed the full TeGeCoach-program (received at least nine coaching calls). In the AT-analysis, patients randomized into the intervention group but who received standard care (did not receive at least 1 coaching call), were treated as if they would have been assigned to the control group.

### Statistical methods

Changes over time between study arms were compared by means of random-effects regression models with the patients’ effect as the random variable. By using the difference-in-differences estimator, observable baseline differences were controlled for. Tests of treatment effects were conducted at a two-sided significance level of 0.05 per model.

In order to examine for the stability of the calculated regression coefficients, differences in observable baseline characteristics between groups were treated with the entropy balancing procedure [32] in a separate analysis. This involves a two-step procedure where in a first step a balancing vector from the entropy balancing procedure is calculated which then was used as a weighting variable in random-effects regression models again. These analyses were intended to function as a sensitivity analysis. Entropy balancing allows a more detailed balancing compared to more conventional processes such as propensity matching since it allows for the balancing of 3 statistical moments (mean, variance and skewness). The entropy balancing procedure was conducted with the following balancing variables: sex, age, status of health insurance, participation in a disease management program, costs of outpatient physician and non-physician services, medical supplies, inpatient treatment, sick pay, medication, rehabilitation and prevention training, as well as membership in health insurance company (TK, KKH, mhplus), daily defined doses (DDD), inpatient treatment days, sick pay days, interaction terms between membership in TK and total costs, mhplus and total costs, TK and DDD, mhplus and DDD, and the 30 conditions that constitute the Elixhauser Index [33]. For the definition of the 30 conditions, only verified ICD diagnoses from pooled outpatient, inpatient and rehabilitation data were used. Statistical analyses were performed using STATA Version 15 (Stata Corp. 2017) [34].

## Results

### Sample characteristics

Sample sizes for the different analyses were *N* = 1685 (IG = 806; CG = 879) for ITT, *N* = 1469 (IG = 590; CG = 879) for mITT, *N* = 1370 (IG = 491; CG = 879) for PP and *N* = 1685 (IG = 590; CG = 1095) for the AT analysis (Fig. 1).

In the ITT sample, 69.0% of patients in the intervention group were males (Table 1). In the control group the proportion of male patients was 67.7%. The mean age was 66.6 years in the intervention group and 66.4 years in the control group. In the intervention group 35.6% of the patients were insured at the KKH, 61.5% at the TK and 2.9% at the mhplus. The corresponding numbers in the control group were 33.4% (KKH), 63.9% (TK) and 2.6% (mhplus). 37.2% of the patients in the intervention group had a status as regular members of a health insurance and 60.9% as pensioners. In the control group, the corresponding proportions were 36.9% members and 60.2% pensioners. In the intervention group, 39.8% took part in a disease management program vs 36.6% in the control group. Further characteristics of the ITT sample can be seen in Table 4. Corresponding characteristics for the mITT, PP and AT samples are shown in the supplemental tables S3, S8 and S13.

**Table 1:** Baseline sample characteristics on socio-demographics and health insurance status of the randomized participants (ITT-sample)

	Total (*n* = 1 685)	Intervention (*n* = 806)	Controls (*n* = 879)
Sex			
Female	534 (31.7)	250 (31.0)	284 (32.3)
Male	1 151 (68.3)	556 (69.0)	595 (67.7)
Age (years)	66.5 (8.6)	66.6 (8.6)	66.4 (8.7)
Health insurance			
KKH	581 (34.5)	287 (35.6)	294 (33.4)
TK	1 058 (62.8)	496 (61.5)	562 (63.9)
mhplus	46 (2.7)	23 (2.9)	23 (2.6)
Status of health insurance			
Regularly insured	624 (37.0)	300 (37.2)	324 (36.9)
Pensioner	1020 (60.5)	491 (60.9)	529 (60.2)
Family	41 (2.4)	15 (1.9)	26 (3.0)
Disease management program	643 (38.2)	321 (39.8)	322 (36.6)

### Unbalanced ITT analysis

#### Health care use

Table 2 lists the estimators of the unbalanced random effects regression models that were carried out for the examined health care use variables. The “constant” indicates the average amount of health care utilization in the control group insured by the KKH at baseline, which was, e.g., 1,722.12 DDDs for medication consumption. The effect parameter labeled “intervention group” indicates the size of the measured differences between the two study groups at baseline. Thus, the average DDD per patient at baseline in the past 12-month period was 71.61 doses higher in the intervention group than in the control group. Although this effect is not significant, this small baseline differences indicates a successful but still improvable randomization due to possible selection bias.

**Table 2:** Regression coefficients of ITT-analysis for health care use (unbalanced)

	DDD	Days in hospital	Sick-pay days
First year	121.80*** (23.32)	1.27*** (0.44)	− 2.69* (1.58)
Second year	257.50*** (30.87)	0.96** (0.46)	− 4.12** (1.84)
Intervention	71.61 (63.42)	0.03 (0.35)	0.82 (2.07)
First year #intervention	21.19 (35.12)	− 0.31 (0.67)	0.41 (2.47)
Second year#intervention	− 3.13 (44.80)	0.27 (0.89)	− 0.21 (2.59)
TK (health insurance)	22.39 (68.13)	0.04 (0.39)	− 0.64 (1.39)
mhplus (health insurance)	− 296.97* (165.79)	− 2.00*** (0.55)	− 0.17 (3.90)
Constant	1,772.12*** (64.03)	3.04*** (0.37)	8.15*** (1.74)
Observations	3856	3856	3856
Patients	1685	1685	1685

Random-effects linear regression model: constant (cost of control group at baseline), intervention (difference from intervention group at baseline), first year (change in cost from baseline in control group), second year (change in cost from baseline in the control group), first year#intervention (DD estimator: cost difference after first year), second year#intervention (DD estimator: cost difference after second year), Robust standard errors in parentheses

***p < 0.01

**p < 0.05

*p < 0.1

According to the interaction terms labelled “first year#intervention group” and “second year#intervention group”, the DDDs increased by 21.19, and declined by 3.13 in the second year. Corresponding DD-estimators for days in hospital were -0.31 in the first year and 0.27 in the second year. For sick pay days, there was an increase of 0.41 days in the first year and a decrease of − 0.21 days in the second year in the intervention group when compared to the controls. All the reported effects were non-significant.

#### Health care costs

Estimators from the unbalanced random effects regression models for the examined health costs are listed in Table 3. The first column contains the results from the model of total costs, which are made up of the individual sum of all previously listed cost variables per patient. DD-estimators of the total costs were €-352 in the first year and €-215 in the second year, i.e. at both times there was a relative, non-significant decrease of the costs in the intervention group compared to the control group. In the individual cost sectors, larger and sometimes opposing effects can be seen in both follow-up years. While the outpatient costs of the intervention group increased by €101 in the first year and decreased by €113 in the second year, the hospital treatment costs decreased by €414 in the first year and by €280 in the second year. The medication costs in the intervention group decreased by €102 in the first year and increased by €116 in the second year. In the case of sick pay costs, a relative increase in the intervention group in both follow-up years by €84 and €93 was observable. All observed cost effects were non-significant.

**Table 3:** Regression coefficients of ITT-analysis for health care costs (unbalanced)

Variables	Total costs	Outpatient physician services	General practicioner	Outpatient non-physician services	Medical supplies	Hospital treatment	Sick pay	Medication	Rehabilitation	Prevention program
First year	1,190.35*** (363.47)	189.07 (125.16)	5.80** (2.37)	24.06** (10.64)	36.92 (25.43)	835.50*** (284.20)	– 104.86 (110.33)	189.24*** (54.73)	27.36 (22.18)	0.19 (0.49)
Second year	1,627.38*** (418.53)	330.45** (143.31)	7.68** (3.16)	48.92*** (13.92)	155.24*** (39.26)	852.82*** (300.14)	– 255.66** (104.44)	448.57*** (144.60)	50.21** (21.28)	– 0.03 (0.78)
Intervention	58.34 (322.59)	7.33 (73.81)	– 8.53 (5.46)	22.73 (20.52)	9.65 (34.25)	43.40 (220.13)	10.66 (126.66)	– 23.61 (82.02)	– 4.61 (15.61)	0.64 (0.81)
First year #intervention	– 351.83 (522.19)	101.39 (181.09)	1.90 (3.03)	– 2.22 (14.99)	– 23.08 (38.98)	– 414.53 (398.26)	84.10 (182.32)	– 102.19 (67.49)	– 10.15 (30.01)	0.08 (0.99)
Second year#intervention	– 215.10 (706.59)	– 113.06 (163.09)	– 5.90 (4.19)	6.88 (21.16)	– 37.57 (54.67)	– 280.00 (494.91)	92.61 (163.18)	116.90 (305.85)	– 9.46 (33.78)	– 0.16 (1.32)
TK (health insurance)	508.61 (323.21)	362.74*** (99.61)	9.62* (5.68)	– 39.79* (22.70)	– 34.42 (35.47)	167.63 (223.48)	34.22 (91.41)	– 61.46 (112.78)	28.05* (14.37)	– 0.44 (0.72)
mhplus (health insurance)	– 1,446.61** (604.49)	– 134.60 (86.19)	42.16* (22.02)	– 133.28*** (25.89)	– 35.06 (59.21)	– 1,059.31*** (353.99)	6.69 (222.54)	– 223.76 (155.12)	– 53.21*** (11.39)	1.82 (2.46)
Constant	4,518.46*** (289.21)	887.50*** (58.94)	32.13*** (5.48)	171.84*** (20.84)	220.08*** (35.98)	1801.08*** (200.64)	436.03*** (104.78)	959.47*** (105.01)	39.11*** (14.96)	2.09*** (0.64)
Observations	3856	3856	3856	3856	3856	3856	3856	3856	3856	3856
Patients	1685	1685	1685	1685	1685	1685	1685	1685	1685	1685

Random-effects linear regression model: constant (cost of control group at baseline), intervention (difference from intervention group at baseline), first year (change in cost from baseline in control group), second year (change in cost from baseline in the control group), first year*intervention (DD estimator: cost difference after first year), second year*intervention (DD estimator: cost difference after second year), Robust standard errors in parentheses

***p < 0.01

**p < 0.05

*p < 0.1

### Entropy balancing (ITT)

Table 4 contains the baseline mean, variance and skewness for the matching variables used in the entropy balancing procedure for the ITT-sample. There is one column for the intervention group and two columns containing the values of the control group pre- and post-balancing, thereby giving an overview about the requirement and success of the balancing. While difference in means disappeared after balancing, there were still notable differences in variance and skewness between the study groups. One extreme example is the skewness of the outpatient costs at baseline, which is 19.07 in the intervention group and 5.81 in the control group (pre balancing), that takes on a value of 4.05 in the control group post-balancing.

**Table 4:** Entropy Balancing ITT N = 1685 (balanced for 1 moment, Interaction Kasse)

	Intervention			Control Pre Balancing			Control Post Balancing		
	Mean	Variance	Skewness	Mean	Variance	Skewness	Mean	Variance	Skewness
Sex: male	0.69	0.21	– 0.82	0.68	0.22	– 0.76	0.69	0.21	– 0.82
Age	66.58	73.56	– 0.49	66.41	74.78	– 0.41	66.58	74.08	– 0.40
Status of health insurance: regularly insured	0.37	0.23	0.53	0.37	0.23	0.54	0.37	0.23	0.53
Status of health insurance: pensioner	0.61	0.24	– 0.45	0.60	0.24	– 0.42	0.61	0.24	– 0.45
Disease management program	0.40	0.24	0.42	0.37	0.23	0.55	0.40	0.24	0.42
Outpatient costs (€)	1114.20	3226447.95	19.07	1115.89	1194001.98	5.81	1114.20	973481.43	4.05
Outpatient non-physician services costs (€)	166.29	207714.28	9.29	142.91	142151.73	6.88	166.29	219727.40	6.52
Medical supplies costs (€)	207.55	555631.91	9.86	197.16	417933.74	6.96	207.55	440005.55	6.43
Hospital costs (€)	1917.41	21844056.39	5.92	1880.54	18695682.66	3.99	1917.41	20500507.16	4.09
Sick pay costs (€)	467.94	7222682.29	7.88	458.09	6197934.63	6.63	467.94	6073330.17	6.58
Medication costs (€)	891.66	2124080.04	6.66	914.32	3566228.58	7.86	891.66	2831768.27	8.15
Rehabilitation costs (€)	50.24	86380.88	6.16	55.65	120122.81	6.87	50.24	103828.36	7.11
Prevention training cost (€)	2.51	335.39	8.74	1.86	213.09	9.09	2.51	294.56	7.84
Total costs	4848.52	48830330.27	4.53	4805.80	37919481.00	2.85	4848.52	38677671.41	2.85
Health insurance company (TK)	0.62	0.24	– 0.47	0.64	0.23	– 0.58	0.62	0.24	– 0.47
Health insurance company (mhplus)	0.03	0.03	5.66	0.03	0.03	5.94	0.03	0.03	5.66
TK*Total costs	3128.92	44038769.25	5.45	3147.14	30572148.29	3.22	3128.92	32208967.38	3.36
mhplus*Total costs	147.13	1824561.95	13.57	74.42	631579.35	16.60	147.13	1828636.44	11.26
Daily defined dose	1849.04	1772058.90	1.15	1778.66	1598490.96	1.10	1849.04	1745343.24	1.09
TK*DDD	1151.79	1971818.34	1.47	1152.85	1730926.40	1.23	1151.79	1847963.68	1.25
mhplus*DDD	44.87	96246.77	8.03	35.12	70161.38	9.23	44.87	99407.26	8.24
Hospital days (number)	3.03	50.96	3.97	3.01	52.58	4.69	3.03	52.45	4.46
Sick pay days	8.57	1935.23	6.12	7.74	1651.42	6.35	8.57	1845.44	6.13
Congestive heart failure	0.14	0.12	2.04	0.13	0.11	2.25	0.14	0.12	2.04
Cardiac arrhythmia	0.19	0.16	1.56	0.19	0.15	1.61	0.19	0.16	1.56
Valvular disease	0.14	0.12	2.10	0.14	0.12	2.12	0.14	0.12	2.10
Pulmonary circulation disorder	0.03	0.03	5.66	0.03	0.02	6.08	0.03	0.03	5.66
Peripheral vascular disease	0.88	0.10	– 2.41	0.87	0.11	– 2.17	0.88	0.10	– 2.41
Hypertension, uncomplicated	0.79	0.17	– 1.42	0.81	0.15	– 1.58	0.79	0.17	– 1.42
Hypertension, complicated	0.16	0.13	1.89	0.16	0.13	1.90	0.16	0.13	1.89
Paralysis	0.02	0.02	6.88	0.02	0.02	6.58	0.02	0.02	6.88
Other neurological disorders	0.03	0.03	5.29	0.03	0.03	5.13	0.03	0.03	5.29
Chronic pulmonary disease	0.25	0.19	1.15	0.26	0.19	1.07	0.25	0.19	1.15
Diabetes, uncomplicated	0.32	0.22	0.78	0.29	0.21	0.94	0.32	0.22	0.78
Diabetes, complicated	0.20	0.16	1.46	0.23	0.17	1.32	0.20	0.16	1.46
Hypothyroidism	0.13	0.12	2.15	0.13	0.11	2.17	0.13	0.12	2.15
Renal failure	0.15	0.13	1.97	0.14	0.12	2.10	0.15	0.13	1.97
Liver disease	0.16	0.14	1.84	0.17	0.14	1.78	0.16	0.14	1.84
Peptic ulcer disease excluding bleeding	0.03	0.03	5.95	0.02	0.02	6.77	0.03	0.03	5.95
Lymphoma	0.01	0.01	12.58	0.01	0.01	11.07	0.01	0.01	12.58
Metastatic cancer	0.01	0.01	8.81	0.01	0.01	8.04	0.01	0.01	8.81
Solid tumor without metastasis	0.11	0.10	2.51	0.11	0.10	2.54	0.11	0.10	2.51
Rheumatoid arthritis/collagen vascular diseases	0.05	0.04	4.34	0.08	0.07	3.13	0.05	0.04	4.34
Coagulopathy	0.06	0.06	3.55	0.05	0.05	4.07	0.06	0.06	3.55
Obesity	0.24	0.18	1.25	0.23	0.18	1.31	0.24	0.18	1.25
Weight loss	0.01	0.01	10.59	0.02	0.02	6.24	0.01	0.01	10.59
Fluid and electrolyte disorders	0.05	0.05	3.92	0.04	0.04	4.87	0.05	0.05	3.92
Blood-loss anemia	0.01	0.01	12.58	0.01	0.01	13.15	0.01	0.01	12.58
Deficiency anemia	0.04	0.04	4.80	0.04	0.04	4.87	0.04	0.04	4.80
Alcohol abuse	0.05	0.05	4.03	0.06	0.06	3.61	0.05	0.05	4.03
Drug abuse	0.01	0.01	12.58	0.01	0.01	11.07	0.01	0.01	12.58
Psychoses	0.00	0.00	20.00	0.00	0.00	14.72	0.00	0.00	20.00
Depression	0.23	0.18	1.30	0.23	0.18	1.31	0.23	0.18	1.30

### Balanced ITT analysis

All baseline differences determined by the "intervention group" effect were almost zero and therefore smaller than in the unbalanced but randomized models (Tables 5 and 6). This implicates a lower degree of potential selection bias. After all, the balanced analyses are largely in accordance with the unbalanced results and thus confirm their reliability.

**Table 5:** Regression coefficients of ITT-analysis for health care use (balanced)

	DDD	Days in hospital	Sick-pay days
First year	95.40*** (25.66)	1.19** (0.48)	– 1.78 (2.16)
Second year	230.02*** (34.55)	0.81* (0.49)	– 5.16** (2.18)
Intervention	0.00 (68.31)	– 0.00 (0.37)	– 0.00 (2.29)
First year #intervention	47.70 (36.71)	– 0.23 (0.70)	– 0.51 (2.89)
Second year#intervention	24.49 (47.40)	0.42 (0.91)	0.85 (2.84)
TK (health insurance)	20.38 (74.21)	0.19 (0.39)	0.06 (1.55)
mhplus (health insurance)	– 223.96 (177.73)	– 1.17 (0.80)	0.02 (4.14)
Constant	1842.89*** (73.36)	2.95*** (0.38)	8.53*** (1.95)
Observations	3856	3856	3856
Patients	1685	1685	1685

Random-effects linear regression model: constant (cost of control group at baseline), intervention (difference from intervention group at baseline), first year (change in cost from baseline in control group), second year (change in cost from baseline in the control group), first year*intervention (DD estimator: cost difference after first year), second year*intervention (DD estimator: cost difference after second year), Robust standard errors in parentheses

***p < 0.01

**p < 0.05

*p < 0.1

**Table 6:** Regression coefficients of ITT-analysis for health care costs (balanced)

	Total costs	Outpatient physician services	General practicioner	Outpatient non– physician services	Medical supplies	Hospital treatment	Sick pay	Medication	Rehabilitation	Prevention program
First year	1013.64*** (385.08)	109.49* (59.45)	5.04*** (1.88)	17.03 (12.32)	25.08 (26.67)	744.44** (307.41)	– 32.95 (126.64)	120.64*** (39.57)	32.15 (22.87)	0.06 (0.56)
Second year	1259.47*** (422.03)	214.82*** (73.14)	8.57*** (2.78)	45.05*** (16.50)	147.59*** (37.44)	722.08** (328.57)	– 287.11** (115.77)	356.33*** (114.63)	64.06*** (23.98)	– 0.41 (0.93)
Intervention	– 0.00 (340.09)	0.00 (71.30)	0.00 (4.97)	0.00 (26.17)	0.00 (35.71)	– 0.00 (241.94)	– 0.00 (132.03)	0.00 (75.17)	0.00 (14.79)	0.00 (0.95)
First year #intervention	– 190.22 (538.74)	181.47 (141.89)	2.65 (2.67)	5.53 (16.12)	– 11.55 (39.77)	– 327.51 (416.33)	8.30 (193.13)	– 35.58 (55.99)	– 14.97 (30.47)	0.19 (1.03)
Second year#intervention	132.37 (708.53)	4.50 (106.70)	– 6.79* (3.91)	11.20 (22.86)	– 29.61 (53.28)	– 150.78 (512.91)	120.81 (170.36)	209.54 (292.96)	– 23.29 (35.52)	0.20 (1.43)
TK (health insurance)	681.77** (311.94)	344.18*** (82.94)	10.75** (5.14)	– 39.29 (28.73)	– 25.63 (36.43)	277.65 (220.03)	66.39 (94.86)	– 27.09 (98.72)	28.27* (14.71)	– 0.35 (0.83)
mhplus (health insurance)	– 430.08 (942.94)	– 70.58 (90.41)	39.00* (21.23)	– 147.51*** (28.28)	– 53.24 (57.39)	– 233.14 (758.85)	25.37 (232.78)	– 159.85 (149.46)	– 51.25*** (11.50)	2.19 (2.99)
Constant	4441.24*** (290.93)	904.42*** (55.68)	22.99*** (4.52)	194.67*** (28.26)	224.84*** (36.72)	1753.20*** (206.32)	426.37*** (107.04)	912.89*** (85.25)	34.31** (13.33)	2.66*** (0.85)
Observations	3856	3856	3856	3856	3856	3856	3856	3856	3856	3856
Patients	1685	1685	1685	1685	1685	1685	1685	1685	1685	1685

Random-effects linear regression model: constant (cost of control group at baseline), intervention (difference from intervention group at baseline), first year (change in cost from baseline in control group), second year (change in cost from baseline in the control group), first year*intervention (DD estimator: cost difference after first year), second year*intervention (DD estimator: cost difference after second year), Robust standard errors in parentheses

***p < 0.01

**p < 0.05

*p < 0.1

### Sensitivity analysis (mITT, PP, AT)

The mITT analysis has a tendency to show slightly larger effects as can be seen in the unbalanced DD-estimators for the total costs with €-534.83 for the first and €-559.49 for the second year (Table S2). A similar pattern can be seen with the balanced results where there was even a change in direction for the DD-estimator in the second year with now €-92.57 for the total costs (Table S5). The unbalanced results for the PP analysis show even larger savings (effects: €-732.98 first year, €-646.98 s year) (Table S7) which is confirmed by the balanced results with €-595.50 in the first and €-226.07 in the second year (Table S10). Finally, the results from the AT analysis confirm the previous results. With unbalanced DD-estimators for total costs of €-630.87 in the first and €-773.56 in the second year, and balanced DD-estimators of €-484.54 in the first and €-277.02 in the second year, we again see a tendency to smaller effects when entropy balancing was applied. As with the ITT analysis the main drivers for the effects on total cost were costs for hospital treatment and medication. This is underlined by the fact that unbalanced DD-estimators for hospital treatment costs in the first year reach the level of significance with €-832.56 in the PP and €-766.46 in the AT analysis. The same holds for medication costs with €-112.44 in the AT analysis. Concerning health care use, another significant unbalanced DD-estimator can be found at first year with -1.23 days in hospital in the AT analysis.

## Discussion

Finding no clear evidence of a significant cost reduction caused by the TeGeCoach program, this health insurance-based clinical trial failed to demonstrate economic benefits of a home-based exercise program with telephone health coaching and exercise monitoring for patients with PAD. This also applies to the investigated health service use variables.

The relative decrease in average total costs of € -351 (first year) or €-215 (second year) per person determined in the unbalanced main ITT analysis was not significant. However, the level of these effects might still be relevant with regard to a possible future implementation of the intervention in a standard care setting. This is underpinned by our finding that the determined cost differences proved to be rather stable with regard to several sensitivity analyses. The cost differences determined with the various sensitivity analyses were also in line with what was to be expected: ITT analysis generally revealed the smallest differences, while PP analysis showed larger differences. This is in accordance with the intention of PP analysis, i.e. to identify treatment effects under optimal conditions, while ITT analysis has the focus on preserving the original randomization and, therefore, is more conservative.

It is further worth noting that there was a tendency for unbalanced DD-estimators of total costs to be smaller in the second year than in the first year. This corresponded largely with similar patterns of hospital treatment costs and medication costs and could be seen in nearly all sensitivity analysis with mITT being the only exception. In case of the hospital treatment costs, this observation is in accordance with the days in hospital that showed smaller DD-estimators in the second year likewise. In the balanced analysis the described pattern is even more pronounced and indicates that the overall cost-reducing effect of the intervention might further diminish after the second year. Furthermore, we observed no signs of a cost shifting effect in the sense that induced by the TeGeCoach hospital treatment costs were shifted to the outpatient sector or vice versa.

A recent RCT by van Reijen [35] analysing cost effectiveness of endovascular revascularization vs. supervised exercise therapy (SET), a treatment being advised for initial intermittent claudication, found costs for SET to be €1852 lower. This is confirmed by another RCT comparing costs of a hospital-based SEP with endovascular revascularization in patients with intermittent claudication that found also significantly higher costs in the revascularization group [36]. Beside the fact that SET has to be applied in a hospital outpatient setting or a physician’s office which distinguishes it from a home-based intervention as the TeGeCoach, our study did not compare the TeGeCoach with revascularization. Therefore, it remains quite speculative, if a HEP such as the TeGeCoach might have similar cost-reducing effects as a SET when being compared to a revascularization group, since our study was conducted in a more naturalistic setting by allowing revascularizations in both study arms. We found no RCT analysing costs associated with PAD treated with an HEP. As consequence, there are few starting points for the discussion of our non-significant study findings. If these are caused by insufficiencies of the TeGeCoach intervention, methodical limitations or if a significant cost reduction is generally unrealistic by means of a HEP for PAD has to remain speculative until other studies shed more light on this topic. However, it has to be mentioned that the conceptualization of the intervention was guided by the aim of achieving positive health effects rather than a cost reduction. Furthermore, it should be pointed out that the study results also mean that we found no evidence for a significant cost increasing effect of the TeGeCoach. This can be seen in favor of the program since significant cost reductions along with positive health benefits are relatively rarely found for new health care technologies.

We are aware that the balancing of randomized controlled trials and the interpretation of the results may seem controversial to some readers. That is why we chose to report the balanced analyses that were previously announced in the study protocol [31] less prominently as another form of sensitivity analysis. Nevertheless, we would argue that at least in our case, backed by the numbers, the randomization was successful though not perfect, when it comes to the mere mean baseline values.

### Strengths and limitations

The generalizability of the results is ensured due to the use of claims data for participant identification, a large sample size, broad inclusion criteria, a wide range of outcomes, long-term participant follow-up and flexible adherence protocols applicable to everyday clinical practice. As a consequence, this might also imply that in order to try to maximize the studies external validity, its internal validity may be hampered by a dilution of the potential treatment effects.

Another limitation is that the 24-month follow-up might have been affected by the circumstances caused by the Covid-19 pandemic. It could be possible that this might have led to a reduced ability or willingness by the respondents to conform to the intervention in order to adhere to the standards of social distancing. If, in consequence, this might have an increasing or decreasing effect on the health care use and costs of the respondents remains speculative.

Furthermore, it has to be mentioned that in German claims data, medication costs associated with hospital treatments are already included in the hospital treatment costs and therefore cannot be analysed separately. Moreover, costs incurring with the intervention were not being considered.

The balancing of the matching variables’ variance and skewness is an often overlooked topic. This is where we saw the greatest potential for a relevant improvement due to the implementation of entropy balancing. Unfortunately, the balancing for the 2nd and 3rd moment was not executable with the data at hand. Although the used sample sizes were rather large, we want to note that the chances to balance for all 3 statistical moments would increase with even larger sample sizes. So, since the sample could only be balanced for the 1^st^ moment, it was not possible to adequately counter the rather large difference in variance and skewness that were observable on outpatient costs before balancing. Nevertheless, balanced results may be considered more reliable as all baseline differences determined by the “intervention group” effect were almost zero and smaller than in the unbalanced models by using the weight vector obtained in the context of entropy balancing, which implies a lower degree of potential selection bias. Therefore, given that the data which inform the balancing contain no differences between study arms that may depend on systematic differences in the underlying data collection process (an assumption which is likely to be valid in claims data), we would argue that the implementation of balancing even in a successful RCT will help to identify estimators that are influenced to an even lesser extent from selection bias.

## Conclusion

We found no clear evidence for a cost reducing effect of the TeGeCoach in terms of significant differences. Nevertheless, most non-significant findings show a tendency for potential cost savings of the program. This is also being supported by the results of the sensitivity analyses, and implicates that there is also no clear evidence for a significant cost increasing effect of the TeGeCoach, which from the perspective of health insurers can be seen as a positive sign for a potential transfer of the program to medical practice.

### Supplementary Information

Below is the link to the electronic supplementary material.

Supplementary file1 (DOCX 131 KB)

## Data Availability

The data that support the findings of this study were used under license from German statutory health insurance companies Kaufmännische Krankenkasse (KKH), Techniker Krankenkasse (TK), and mhplus Krankenkasse for the current study. Due to strict data protection rules according to Fünftes Buch Sozialgesetzbuch (SGB V), the data cannot be made publicly accessible.
