# Supplementary material for: The effect of telephone health coaching and remote exercise monitoring for peripheral artery disease (TeGeCoach) on health care cost and utilization: results of a randomized controlled trial
Source: Eur J Health Econ. 2023 Jul 10;25(4):615–29. doi: 10.1007/s10198-023-01616-4 (PMC11136827; doi:10.1007/s10198-023-01616-4)
Supplement: Supplementary file 1 — Supplementary file1 (DOCX 131 KB) [file 10198_2023_1616_MOESM1_ESM.docx]

Table S1 Regression coefficients of mITT-analysis for health care use (unbalanced)

|  |  |  |  |
| --- | --- | --- | --- |
|  | DDD | Days in hospital | Sick-pay days |
|  |  |  |  |
| First year | 121.99*** | 1.25*** | -2.69* |
|  | (23.33) | (0.44) | (1.58) |
| Second year | 257.68*** | 0.95** | -4.13** |
|  | (30.88) | (0.46) | (1.84) |
| Intervention | 35.65 | -0.10 | 1.82 |
|  | (67.97) | (0.38) | (2.36) |
| First year #intervention | 30.30 | -0.87 | 0.44 |
|  | (36.67) | (0.63) | (2.75) |
| Second year#intervention | 7.77 | -0.50 | -1.11 |
|  | (46.20) | (0.69) | (2.81) |
| TK (health insurance) | 13.11 | 0.33 | -0.73 |
|  | (75.04) | (0.33) | (1.53) |
| mhplus (health insurance) | -179.06 | -1.86*** | -1.23 |
|  | (179.42) | (0.52) | (4.12) |
| Constant | 1,774.97*** | 2.85*** | 8.24*** |
|  | (67.78) | (0.35) | (1.81) |
|  |  |  |  |
| Observations | 3,511 | 3,511 | 3,511 |
| Patients | 1,469 | 1,469 | 1,469 |

Random-effects linear regression model: constant (cost of control group at baseline), intervention (difference from intervention group at baseline), first year (change in cost from baseline in control group), second year (change in cost from baseline in the control group), first year*intervention (DD estimator: cost difference after first year), second year*intervention (DD estimator: cost difference after second year), Robust standard errors in parentheses; ***p<0.01, **p<0.05, *p<0.1

Table S2 Regression coefficients of mITT for health care costs (unbalanced)

|  |  |  |  |  |  |  |  |  |  |  |
| --- | --- | --- | --- | --- | --- | --- | --- | --- | --- | --- |
|  | Total costs | Outpatient physician services | General practicioner | Outpatient non-physician services | Medical supplies | Hospital treatment | Sick pay | Medication | Rehabilitation | Prevention program |
|  |  |  |  |  |  |  |  |  |  |  |
| First year | 1,178.38*** | 188.36 | 5.79** | 24.14** | 37.53 | 826.18*** | -104.54 | 189.46*** | 26.84 | 0.18 |
|  | (362.91) | (124.81) | (2.37) | (10.70) | (25.38) | (284.21) | (110.14) | (54.62) | (22.16) | (0.49) |
| Second year | 1,621.29*** | 330.08** | 7.67** | 48.88*** | 155.71*** | 847.50*** | -256.18** | 449.18*** | 50.01** | -0.04 |
|  | (418.16) | (143.01) | (3.16) | (13.96) | (39.19) | (300.21) | (104.58) | (144.48) | (21.27) | (0.78) |
| Intervention | 99.73 | 15.60 | -8.40 | 25.56 | 25.08 | -15.86 | 75.02 | -5.12 | -10.98 | 1.09 |
|  | (369.83) | (89.99) | (5.93) | (19.65) | (40.23) | (245.01) | (147.07) | (91.47) | (16.59) | (0.98) |
| First year #intervention | -534.83 | 139.16 | 2.99 | -2.30 | -30.66 | -652.83 | 98.40 | -114.13 | -0.58 | 0.08 |
|  | (543.86) | (194.12) | (3.19) | (15.65) | (42.17) | (403.83) | (203.92) | (69.79) | (31.31) | (1.14) |
| Second year#intervention | -559.49 | -81.82 | -5.51 | 5.09 | -47.91 | -589.88 | 54.78 | 133.20 | -17.21 | -1.18 |
|  | (695.29) | (168.21) | (4.44) | (21.32) | (57.41) | (446.74) | (181.68) | (337.27) | (31.60) | (1.30) |
| TK (health insurance) | 633.43* | 376.51*** | 10.94* | -50.20** | -44.79 | 322.17 | 20.59 | -73.16 | 34.67** | -0.55 |
|  | (331.20) | (107.67) | (6.22) | (24.56) | (40.52) | (207.99) | (102.82) | (125.40) | (14.44) | (0.81) |
| mhplus (health insurance) | -1,396.95** | -98.66 | 33.16 | -141.08*** | -70.89 | -986.28*** | -70.75 | -189.22 | -47.22*** | 1.56 |
|  | (665.39) | (95.32) | (22.16) | (29.37) | (60.45) | (367.43) | (235.83) | (173.76) | (11.21) | (2.77) |
| Constant | 4,437.36*** | 877.75*** | 31.52*** | 178.70*** | 227.65*** | 1,700.36*** | 446.77*** | 966.05*** | 34.72** | 2.17*** |
|  | (292.08) | (61.64) | (5.73) | (22.04) | (38.96) | (191.90) | (109.54) | (112.32) | (14.99) | (0.68) |
|  |  |  |  |  |  |  |  |  |  |  |
| Observations | 3,511 | 3,511 | 3,511 | 3,511 | 3,511 | 3,511 | 3,511 | 3,511 | 3,511 | 3,511 |
| Patients | 1,469 | 1,469 | 1,469 | 1,469 | 1,469 | 1,469 | 1,469 | 1,469 | 1,469 | 1,469 |

Random-effects linear regression model: constant (cost of control group at baseline), intervention (difference from intervention group at baseline), first year (change in cost from baseline in control group), second year (change in cost from baseline in the control group), first year*intervention (DD estimator: cost difference after first year), second year*intervention (DD estimator: cost difference after second year), Robust standard errors in parentheses; ***p<0.01, **p<0.05, *p<0.1

Table S3 Entropy Balancing mITT mit Ausreißern N=1469 (balanced for 1st moment, Interaction health insurance company)

|  | **Intervention** | | | **Control Pre Balancing** | | | **Control Post Balancing** | | |
| --- | --- | --- | --- | --- | --- | --- | --- | --- | --- |
|  | **Mean** | **Variance** | **Skewness** | **Mean** | **Variance** | **Skewness** | **Mean** | **Variance** | **Skewness** |
| **Sex (male)** | 0.71 | 0.21 | -0.93 | 0.68 | 0.22 | -0.76 | 0.71 | 0.21 | -0.93 |
| **Age** | 66.48 | 69.77 | -0.35 | 66.41 | 74.78 | -0.41 | 66.48 | 73.45 | -0.38 |
| **Status of health insurance: regularly insured** | 0.38 | 0.24 | 0.48 | 0.37 | 0.23 | 0.54 | 0.38 | 0.24 | 0.48 |
| **Status of health insurance: pensioner** | 0.60 | 0.24 | -0.41 | 0.60 | 0.24 | -0.42 | 0.60 | 0.24 | -0.41 |
| **Disease management program** | 0.38 | 0.24 | 0.48 | 0.37 | 0.23 | 0.55 | 0.38 | 0.24 | 0.48 |
| **Outpatient costs (€)** | 1149.60 | 4159109.78 | 17.72 | 1115.89 | 1194001.98 | 5.81 | 1149.60 | 1201947.71 | 5.01 |
| **Outpatient non-physician services costs (€)** | 165.65 | 132451.13 | 4.12 | 142.91 | 142151.73 | 6.88 | 165.65 | 216064.58 | 6.35 |
| **Medical supplies costs (€)** | 219.87 | 688227.77 | 9.53 | 197.16 | 417933.74 | 6.96 | 219.87 | 517756.77 | 6.27 |
| **Hospital costs (€)** | 1877.78 | 23097261.39 | 6.57 | 1880.54 | 18695682.66 | 3.99 | 1877.78 | 19508363.98 | 4.09 |
| **Sick pay costs (€)** | 533.93 | 8665578.33 | 7.54 | 458.09 | 6197934.63 | 6.63 | 533.93 | 7198588.28 | 6.19 |
| **Medication costs (€)** | 905.13 | 2576590.99 | 6.67 | 914.32 | 3566228.58 | 7.86 | 905.13 | 3258533.39 | 7.74 |
| **Rehabilitation costs (€)** | 46.24 | 82872.81 | 6.61 | 55.65 | 120122.81 | 6.87 | 46.24 | 93058.44 | 7.29 |
| **Prevention training cost (€)** | 2.93 | 404.47 | 8.16 | 1.86 | 213.09 | 9.09 | 2.93 | 357.68 | 7.34 |
| **Total costs** | 4932.72 | 55879424.41 | 4.71 | 4805.80 | 37919481.00 | 2.85 | 4932.72 | 39820946.58 | 2.74 |
| **Health insurance company (TK)** | 0.69 | 0.21 | -0.81 | 0.64 | 0.23 | -0.58 | 0.69 | 0.21 | -0.81 |
| **Health insurance company (mhplus)** | 0.03 | 0.03 | 5.63 | 0.03 | 0.03 | 5.94 | 0.03 | 0.03 | 5.63 |
| **TK*Total costs** | 3530.73 | 52172442.85 | 5.34 | 3147.14 | 30572148.29 | 3.22 | 3530.73 | 35506239.33 | 3.12 |
| **mhplus*Total costs** | 164.55 | 2295630.00 | 12.76 | 74.42 | 631579.35 | 16.60 | 164.55 | 1977237.99 | 10.31 |
| **Daily defined dose** | 1814.48 | 1656676.90 | 1.16 | 1778.66 | 1598490.96 | 1.10 | 1814.48 | 1676756.49 | 1.07 |
| **TK*DDD** | 1252.68 | 1841173.03 | 1.30 | 1152.85 | 1730926.40 | 1.23 | 1252.68 | 1810064.07 | 1.12 |
| **mhplus*DDD** | 52.76 | 118674.96 | 7.42 | 35.12 | 70161.38 | 9.23 | 52.76 | 130556.49 | 7.87 |
| **Hospital days (number)** | 2.92 | 49.96 | 4.15 | 3.01 | 52.58 | 4.69 | 2.92 | 46.44 | 4.35 |
| **Sick pay days** | 9.52 | 2178.49 | 5.91 | 7.74 | 1651.42 | 6.35 | 9.52 | 2037.75 | 5.76 |
| **Congestive heart failure** | 0.13 | 0.11 | 2.17 | 0.13 | 0.11 | 2.25 | 0.13 | 0.11 | 2.17 |
| **Cardiac arrhythmia** | 0.17 | 0.14 | 1.71 | 0.19 | 0.15 | 1.61 | 0.17 | 0.14 | 1.71 |
| **Valvular disease** | 0.14 | 0.12 | 2.07 | 0.14 | 0.12 | 2.12 | 0.14 | 0.12 | 2.07 |
| **Pulmonary circulation disorder** | 0.03 | 0.03 | 5.82 | 0.03 | 0.02 | 6.08 | 0.03 | 0.03 | 5.82 |
| **Peripheral vascular disease** | 0.91 | 0.08 | -2.94 | 0.87 | 0.11 | -2.17 | 0.91 | 0.08 | -2.94 |
| **Hypertension, uncomplicated** | 0.79 | 0.17 | -1.41 | 0.81 | 0.15 | -1.58 | 0.79 | 0.17 | -1.41 |
| **Hypertension, complicated** | 0.16 | 0.13 | 1.90 | 0.16 | 0.13 | 1.90 | 0.16 | 0.13 | 1.90 |
| **Paralysis** | 0.02 | 0.02 | 7.91 | 0.02 | 0.02 | 6.58 | 0.02 | 0.02 | 7.91 |
| **Other neurological disorders** | 0.03 | 0.03 | 5.63 | 0.03 | 0.03 | 5.13 | 0.03 | 0.03 | 5.63 |
| **Chronic pulmonary disease** | 0.25 | 0.19 | 1.18 | 0.26 | 0.19 | 1.07 | 0.25 | 0.19 | 1.18 |
| **Diabetes, uncomplicated** | 0.31 | 0.21 | 0.82 | 0.29 | 0.21 | 0.94 | 0.31 | 0.21 | 0.82 |
| **Diabetes, complicated** | 0.19 | 0.16 | 1.57 | 0.23 | 0.17 | 1.32 | 0.19 | 0.16 | 1.57 |
| **Hypothyroidism** | 0.13 | 0.12 | 2.15 | 0.13 | 0.11 | 2.17 | 0.13 | 0.12 | 2.15 |
| **Renal failure** | 0.14 | 0.12 | 2.05 | 0.14 | 0.12 | 2.10 | 0.14 | 0.12 | 2.05 |
| **Liver disease** | 0.15 | 0.13 | 1.93 | 0.17 | 0.14 | 1.78 | 0.15 | 0.13 | 1.93 |
| **Peptic ulcer disease excluding bleeding** | 0.02 | 0.02 | 6.26 | 0.02 | 0.02 | 6.77 | 0.02 | 0.02 | 6.26 |
| **Lymphoma** | 0.00 | 0.00 | 17.09 | 0.01 | 0.01 | 11.07 | 0.00 | 0.00 | 17.09 |
| **Metastatic cancer** | 0.01 | 0.01 | 9.02 | 0.01 | 0.01 | 8.04 | 0.01 | 0.01 | 9.02 |
| **Solid tumor without metastasis** | 0.10 | 0.09 | 2.70 | 0.11 | 0.10 | 2.54 | 0.10 | 0.09 | 2.70 |
| **Rheumatoid arthritis/collagen vascular diseases** | 0.04 | 0.04 | 4.76 | 0.08 | 0.07 | 3.13 | 0.04 | 0.04 | 4.76 |
| **Coagulopathy** | 0.06 | 0.05 | 3.86 | 0.05 | 0.05 | 4.07 | 0.06 | 0.05 | 3.86 |
| **Obesity** | 0.22 | 0.17 | 1.35 | 0.23 | 0.18 | 1.31 | 0.22 | 0.17 | 1.35 |
| **Weight loss** | 0.01 | 0.01 | 13.92 | 0.02 | 0.02 | 6.24 | 0.01 | 0.01 | 13.92 |
| **Fluid and electrolyte disorders** | 0.05 | 0.05 | 4.01 | 0.04 | 0.04 | 4.87 | 0.05 | 0.05 | 4.01 |
| **Blood-loss anemia** | 0.01 | 0.01 | 12.02 | 0.01 | 0.01 | 13.15 | 0.01 | 0.01 | 12.02 |
| **Deficiency anemia** | 0.03 | 0.03 | 5.30 | 0.04 | 0.04 | 4.87 | 0.03 | 0.03 | 5.30 |
| **Alcohol abuse** | 0.04 | 0.04 | 4.44 | 0.06 | 0.06 | 3.61 | 0.04 | 0.04 | 4.44 |
| **Drug abuse** | 0.01 | 0.01 | 12.02 | 0.01 | 0.01 | 11.07 | 0.01 | 0.01 | 12.02 |
| **Psychoses** | 0.00 | 0.00 | 24.23 | 0.00 | 0.00 | 14.72 | 0.00 | 0.00 | 24.23 |
| **Depression** | 0.21 | 0.16 | 1.45 | 0.23 | 0.18 | 1.31 | 0.21 | 0.16 | 1.45 |

Table S4 Regression coefficients of mITT-analysis for health care use (balanced)

|  |  |  |  |
| --- | --- | --- | --- |
|  | DDD | Days in hospital | Sick-pay days |
|  |  |  |  |
| First year | 96.29*** | 1.28*** | -1.75 |
|  | (24.87) | (0.49) | (2.47) |
| Second year | 213.28*** | 0.76* | -6.16** |
|  | (34.44) | (0.45) | (2.46) |
| Intervention | 0.00 | -0.00 | -0.00 |
|  | (72.50) | (0.39) | (2.76) |
| First year #intervention | 56.28 | -0.91 | -0.44 |
|  | (37.66) | (0.67) | (3.34) |
| Second year#intervention | 52.47 | -0.31 | 1.01 |
|  | (48.64) | (0.69) | (3.24) |
| TK (health insurance) | -1.45 | 0.54* | 0.09 |
|  | (83.18) | (0.31) | (1.86) |
| mhplus (health insurance) | 76.85 | -0.68 | -0.37 |
|  | (212.16) | (0.78) | (5.23) |
| Constant | 1,813.26*** | 2.56*** | 9.47*** |
|  | (79.02) | (0.34) | (2.28) |
|  |  |  |  |
| Observations | 3,511 | 3,511 | 3,511 |
| Patients | 1,469 | 1,469 | 1,469 |

Random-effects linear regression model: constant (cost of control group at baseline), intervention (difference from intervention group at baseline), first year (change in cost from baseline in control group), second year (change in cost from baseline in the control group), first year*intervention (DD estimator: cost difference after first year), second year*intervention (DD estimator: cost difference after second year), Robust standard errors in parentheses; ***p<0.01, **p<0.05, *p<0.1

Table S5 Regression coefficients of mITT-analysis for health care costs (balanced)

|  |  |  |  |  |  |  |  |  |  |  |
| --- | --- | --- | --- | --- | --- | --- | --- | --- | --- | --- |
|  | Total costs | Outpatient physician services | General practicioner | Outpatient non-physician services | Medical supplies | Hospital treatment | Sick pay | Medication | Rehabilitation | Prevention program |
|  |  |  |  |  |  |  |  |  |  |  |
| First year | 1,084.96*** | 151.53 | 4.57** | 10.93 | 26.39 | 784.86** | -23.80 | 87.98** | 44.03* | -0.03 |
|  | (413.93) | (102.74) | (1.87) | (12.41) | (27.15) | (311.72) | (150.64) | (40.76) | (24.52) | (0.59) |
| Second year | 1,143.11*** | 254.16** | 9.09*** | 36.78** | 147.20*** | 734.13** | -343.28** | 251.95*** | 63.93*** | -0.72 |
|  | (433.03) | (118.83) | (3.08) | (15.55) | (38.11) | (326.03) | (143.05) | (79.37) | (23.28) | (0.96) |
| Intervention | -0.00 | 0.00 | 0.00 | 0.00 | 0.00 | -0.00 | -0.00 | 0.00 | 0.00 | 0.00 |
|  | (399.89) | (93.14) | (5.61) | (25.79) | (45.57) | (265.47) | (168.94) | (93.85) | (15.67) | (1.20) |
| First year #intervention | -451.25 | 176.25 | 4.21 | 11.52 | -17.74 | -611.78 | 15.20 | -14.27 | -17.62 | 0.29 |
|  | (580.19) | (179.90) | (2.84) | (16.85) | (43.11) | (424.27) | (228.65) | (59.46) | (33.01) | (1.19) |
| Second year#intervention | -92.57 | -3.48 | -6.93 | 17.47 | -36.60 | -472.02 | 139.25 | 330.87 | -31.02 | -0.53 |
|  | (704.34) | (148.44) | (4.38) | (22.35) | (56.27) | (464.63) | (205.89) | (314.88) | (32.94) | (1.42) |
| TK (health insurance) | 837.42** | 373.98*** | 13.44** | -52.38* | -49.07 | 418.12** | 61.54 | -26.34 | 31.94** | -0.66 |
|  | (330.51) | (106.39) | (5.88) | (30.27) | (48.34) | (196.63) | (122.64) | (117.42) | (15.16) | (1.09) |
| mhplus (health insurance) | 56.77 | 40.99 | 34.23 | -160.65*** | -109.61* | 5.40 | -23.72 | 3.20 | -43.40*** | 1.91 |
|  | (1,004.42) | (130.72) | (23.21) | (31.16) | (62.45) | (715.31) | (294.45) | (188.06) | (11.83) | (3.63) |
| Constant | 4,354.83*** | 891.07*** | 21.37*** | 206.32*** | 256.79*** | 1,589.90*** | 492.26*** | 923.17*** | 25.51* | 3.32*** |
|  | (316.61) | (73.38) | (5.11) | (29.55) | (48.13) | (199.84) | (134.82) | (103.00) | (13.25) | (1.12) |
|  |  |  |  |  |  |  |  |  |  |  |
| Observations | 3,511 | 3,511 | 3,511 | 3,511 | 3,511 | 3,511 | 3,511 | 3,511 | 3,511 | 3,511 |
| Patients | 1,469 | 1,469 | 1,469 | 1,469 | 1,469 | 1,469 | 1,469 | 1,469 | 1,469 | 1,469 |

Random-effects linear regression model: constant (cost of control group at baseline), intervention (difference from intervention group at baseline), first year (change in cost from baseline in control group), second year (change in cost from baseline in the control group), first year*intervention (DD estimator: cost difference after first year), second year*intervention (DD estimator: cost difference after second year), Robust standard errors in parentheses; ***p<0.01, **p<0.05, *p<0.1

Table S6 Regression coefficients of Per Protocol analysis for health care use (unbalanced)

|  |  |  |  |
| --- | --- | --- | --- |
|  | DDD | Days in hospital | Sick-pay days |
|  |  |  |  |
| First year | 121.98*** | 1.25*** | -2.68* |
|  | (23.33) | (0.44) | (1.58) |
| Second year | 257.65*** | 0.95** | -4.11** |
|  | (30.88) | (0.46) | (1.83) |
| Intervention | 21.87 | -0.01 | 0.88 |
|  | (71.89) | (0.41) | (2.43) |
| First year #intervention | 33.89 | -1.04 | 0.80 |
|  | (37.14) | (0.65) | (2.77) |
| Second year#intervention | 13.09 | -0.65 | -0.54 |
|  | (46.68) | (0.71) | (2.89) |
| TK (health insurance) | 21.38 | 0.34 | -0.95 |
|  | (77.65) | (0.34) | (1.56) |
| mhplus (health insurance) | -186.56 | -1.94*** | -3.50 |
|  | (199.51) | (0.55) | (3.35) |
| Constant | 1,769.87*** | 2.84*** | 8.44*** |
|  | (69.28) | (0.35) | (1.82) |
|  |  |  |  |
| Observations | 3,388 | 3,388 | 3,388 |
| Patients | 1,370 | 1,370 | 1,370 |

Random-effects linear regression model: constant (cost of control group at baseline), intervention (difference from intervention group at baseline), first year (change in cost from baseline in control group), second year (change in cost from baseline in the control group), first year*intervention (DD estimator: cost difference after first year), second year*intervention (DD estimator: cost difference after second year), Robust standard errors in parentheses; ***p<0.01, **p<0.05, *p<0.1

Table S7 Regression coefficients of Per Protocol analysis for health care costs (unbalanced)

|  |  |  |  |  |  |  |  |  |  |  |
| --- | --- | --- | --- | --- | --- | --- | --- | --- | --- | --- |
|  | Total costs | Outpatient physician services | General practicioner | Outpatient non-physician services | Medical supplies | Hospital treatment | Sick pay | Medication | Rehabilitation | Prevention program |
|  |  |  |  |  |  |  |  |  |  |  |
| First year | 1,175.10*** | 187.65 | 5.82** | 24.17** | 37.43 | 823.36*** | -103.85 | 189.07*** | 26.23 | 0.17 |
|  | (362.75) | (124.62) | (2.37) | (10.70) | (25.37) | (284.23) | (110.04) | (54.55) | (22.15) | (0.49) |
| Second year | 1,618.65*** | 329.66** | 7.67** | 48.91*** | 155.71*** | 845.35*** | -256.27** | 449.13*** | 49.67** | -0.05 |
|  | (418.04) | (142.83) | (3.16) | (13.97) | (39.17) | (300.22) | (104.56) | (144.41) | (21.27) | (0.78) |
| Intervention | 154.74 | 30.50 | -10.48* | 20.36 | 8.47 | 58.08 | 52.34 | 3.14 | -7.11 | 0.86 |
|  | (405.23) | (103.72) | (6.12) | (20.47) | (42.79) | (271.09) | (155.42) | (99.58) | (17.90) | (1.01) |
| First year #intervention | -732.98 | 149.67 | 3.71 | -2.75 | -21.27 | -832.56** | 57.90 | -113.74 | -18.33 | 0.36 |
|  | (545.19) | (194.10) | (3.20) | (15.56) | (43.23) | (415.09) | (198.38) | (70.37) | (30.27) | (1.16) |
| Second year#intervention | -646.98 | -81.28 | -4.96 | 6.80 | -50.81 | -696.88 | 53.44 | 139.87 | -19.32 | -0.93 |
|  | (713.38) | (168.75) | (4.51) | (21.48) | (56.61) | (460.87) | (188.90) | (344.99) | (32.58) | (1.33) |
| TK (health insurance) | 663.07* | 394.48*** | 8.53 | -51.32** | -39.55 | 356.04* | 1.89 | -73.03 | 43.79*** | -0.29 |
|  | (341.61) | (112.42) | (6.56) | (25.71) | (41.80) | (212.92) | (105.85) | (131.50) | (13.63) | (0.80) |
| mhplus (health insurance) | -1,648.27*** | -85.87 | 26.01 | -135.54*** | -51.12 | -1,084.21*** | -213.26 | -206.66 | -41.61*** | 1.13 |
|  | (607.80) | (103.16) | (21.70) | (31.65) | (65.14) | (377.54) | (187.24) | (182.92) | (10.04) | (2.84) |
| Constant | 4,424.99*** | 865.93*** | 33.25*** | 179.27*** | 223.78*** | 1,681.27*** | 462.46*** | 966.43*** | 28.74** | 2.01*** |
|  | (296.75) | (63.79) | (5.90) | (22.71) | (39.86) | (193.84) | (110.87) | (115.81) | (14.65) | (0.67) |
|  |  |  |  |  |  |  |  |  |  |  |
| Observations | 3,388 | 3,388 | 3,388 | 3,388 | 3,388 | 3,388 | 3,388 | 3,388 | 3,388 | 3,388 |
| Patients | 1,370 | 1,370 | 1,370 | 1,370 | 1,370 | 1,370 | 1,370 | 1,370 | 1,370 | 1,370 |

Random-effects linear regression model: constant (cost of control group at baseline), intervention (difference from intervention group at baseline), first year (change in cost from baseline in control group), second year (change in cost from baseline in the control group), first year*intervention (DD estimator: cost difference after first year), second year*intervention (DD estimator: cost difference after second year), Robust standard errors in parentheses; ***p<0.01, **p<0.05, *p<0.1

Table S8 Entropy Balancing Per Protocol N=1368 (balanced for 1st moment, Interaction health insurance company)

|  | **Intervention** | | | **Control Pre Balancing** | | | **Control Post Balancing** | | |
| --- | --- | --- | --- | --- | --- | --- | --- | --- | --- |
|  | **Mean** | **Variance** | **Skewness** | **Mean** | **Variance** | **Skewness** | **Mean** | **Variance** | **Skewness** |
| **Sex (male)** | 0.73 | 0.20 | -1.01 | 0.68 | 0.22 | -0.76 | 0.73 | 0.20 | -1.01 |
| **Age** | 66.71 | 67.80 | -0.34 | 66.41 | 74.78 | -0.41 | 66.71 | 72.88 | -0.43 |
| **Status of health insurance: regularly insured** | 0.37 | 0.23 | 0.54 | 0.37 | 0.23 | 0.54 | 0.37 | 0.23 | 0.54 |
| **Status of health insurance: pensioner** | 0.62 | 0.24 | -0.49 | 0.60 | 0.24 | -0.42 | 0.62 | 0.24 | -0.49 |
| **Disease management program** | 0.39 | 0.24 | 0.46 | 0.37 | 0.23 | 0.55 | 0.39 | 0.24 | 0.46 |
| **Outpatient costs (€)** | 1175.52 | 4900816.22 | 16.61 | 1115.89 | 1194001.98 | 5.81 | 1175.52 | 1376998.21 | 5.43 |
| **Outpatient non-physician services costs (€)** | 159.73 | 124972.67 | 4.19 | 142.91 | 142151.73 | 6.88 | 159.73 | 193479.65 | 6.35 |
| **Medical supplies costs (€)** | 202.81 | 675924.64 | 10.41 | 197.16 | 417933.74 | 6.96 | 202.81 | 437032.56 | 6.46 |
| **Hospital costs (€)** | 1966.64 | 25925052.93 | 6.52 | 1880.54 | 18695682.66 | 3.99 | 1966.64 | 21140023.79 | 4.04 |
| **Sick pay costs (€)** | 510.93 | 8405429.25 | 7.73 | 458.09 | 6197934.63 | 6.63 | 510.93 | 7173815.30 | 6.43 |
| **Medication costs (€)** | 912.45 | 2877914.81 | 6.66 | 914.32 | 3566228.58 | 7.86 | 912.45 | 3289840.01 | 7.59 |
| **Rehabilitation costs (€)** | 51.83 | 92664.09 | 6.25 | 55.65 | 120122.81 | 6.87 | 51.83 | 103624.82 | 6.88 |
| **Prevention training cost (€)** | 2.70 | 370.25 | 8.31 | 1.86 | 213.09 | 9.09 | 2.70 | 324.46 | 7.60 |
| **Total costs** | 5012.10 | 60117425.23 | 4.74 | 4805.80 | 37919481.00 | 2.85 | 5012.10 | 41597222.69 | 2.74 |
| **Health insurance company (TK)** | 0.71 | 0.21 | -0.94 | 0.64 | 0.23 | -0.58 | 0.71 | 0.20 | -0.94 |
| **Health insurance company (mhplus)** | 0.02 | 0.02 | 6.16 | 0.03 | 0.03 | 5.94 | 0.02 | 0.02 | 6.16 |
| **TK*Total costs** | 3705.03 | 56373718.05 | 5.30 | 3147.14 | 30572148.29 | 3.22 | 3705.04 | 37633668.86 | 3.07 |
| **mhplus*Total costs** | 126.88 | 1409015.99 | 13.82 | 74.42 | 631579.35 | 16.60 | 126.88 | 1391549.48 | 11.53 |
| **Daily defined dose** | 1802.42 | 1647278.22 | 1.20 | 1778.66 | 1598490.96 | 1.10 | 1802.42 | 1660644.75 | 1.04 |
| **TK*DDD** | 1292.43 | 1838083.22 | 1.27 | 1152.85 | 1730926.40 | 1.23 | 1292.43 | 1818105.13 | 1.09 |
| **mhplus*DDD** | 45.77 | 110330.63 | 8.29 | 35.12 | 70161.38 | 9.23 | 45.77 | 116451.01 | 8.54 |
| **Hospital days (number)** | 3.02 | 53.82 | 4.13 | 3.01 | 52.58 | 4.69 | 3.02 | 49.02 | 4.31 |
| **Sick pay days** | 8.55 | 1971.91 | 6.40 | 7.74 | 1651.42 | 6.35 | 8.55 | 1770.50 | 5.98 |
| **Congestive heart failure** | 0.13 | 0.12 | 2.14 | 0.13 | 0.11 | 2.25 | 0.13 | 0.12 | 2.14 |
| **Cardiac arrhythmia** | 0.19 | 0.15 | 1.62 | 0.19 | 0.15 | 1.61 | 0.19 | 0.15 | 1.62 |
| **Valvular disease** | 0.14 | 0.12 | 2.04 | 0.14 | 0.12 | 2.12 | 0.14 | 0.12 | 2.04 |
| **Pulmonary circulation disorder** | 0.03 | 0.03 | 5.67 | 0.03 | 0.02 | 6.08 | 0.03 | 0.03 | 5.67 |
| **Peripheral vascular disease** | 0.91 | 0.08 | -2.92 | 0.87 | 0.11 | -2.17 | 0.91 | 0.08 | -2.92 |
| **Hypertension, uncomplicated** | 0.78 | 0.17 | -1.34 | 0.81 | 0.15 | -1.58 | 0.78 | 0.17 | -1.34 |
| **Hypertension, complicated** | 0.16 | 0.14 | 1.85 | 0.16 | 0.13 | 1.90 | 0.16 | 0.14 | 1.85 |
| **Paralysis** | 0.01 | 0.01 | 8.19 | 0.02 | 0.02 | 6.58 | 0.01 | 0.01 | 8.19 |
| **Other neurological disorders** | 0.02 | 0.02 | 6.16 | 0.03 | 0.03 | 5.13 | 0.02 | 0.02 | 6.16 |
| **Chronic pulmonary disease** | 0.25 | 0.19 | 1.16 | 0.26 | 0.19 | 1.07 | 0.25 | 0.19 | 1.16 |
| **Diabetes, uncomplicated** | 0.31 | 0.21 | 0.84 | 0.29 | 0.21 | 0.94 | 0.31 | 0.21 | 0.84 |
| **Diabetes, complicated** | 0.19 | 0.16 | 1.55 | 0.23 | 0.17 | 1.32 | 0.19 | 0.16 | 1.55 |
| **Hypothyroidism** | 0.13 | 0.11 | 2.22 | 0.13 | 0.11 | 2.17 | 0.13 | 0.11 | 2.22 |
| **Renal failure** | 0.14 | 0.12 | 2.04 | 0.14 | 0.12 | 2.10 | 0.14 | 0.12 | 2.04 |
| **Liver disease** | 0.14 | 0.12 | 2.04 | 0.17 | 0.14 | 1.78 | 0.14 | 0.12 | 2.04 |
| **Peptic ulcer disease excluding bleeding** | 0.03 | 0.03 | 5.90 | 0.02 | 0.02 | 6.77 | 0.03 | 0.03 | 5.90 |
| **Lymphoma** | 0.00 | 0.00 | 15.57 | 0.01 | 0.01 | 11.07 | 0.00 | 0.00 | 15.57 |
| **Metastatic cancer** | 0.01 | 0.01 | 8.88 | 0.01 | 0.01 | 8.04 | 0.01 | 0.01 | 8.88 |
| **Solid tumor without metastasis** | 0.10 | 0.09 | 2.60 | 0.11 | 0.10 | 2.54 | 0.10 | 0.09 | 2.60 |
| **Rheumatoid arthritis/collagen vascular diseases** | 0.04 | 0.04 | 4.93 | 0.08 | 0.07 | 3.13 | 0.04 | 0.04 | 4.93 |
| **Coagulopathy** | 0.06 | 0.05 | 3.82 | 0.05 | 0.05 | 4.07 | 0.06 | 0.05 | 3.82 |
| **Obesity** | 0.21 | 0.17 | 1.40 | 0.23 | 0.18 | 1.31 | 0.21 | 0.17 | 1.40 |
| **Weight loss** | 0.01 | 0.01 | 12.68 | 0.02 | 0.02 | 6.24 | 0.01 | 0.01 | 12.68 |
| **Fluid and electrolyte disorders** | 0.05 | 0.05 | 3.90 | 0.04 | 0.04 | 4.87 | 0.05 | 0.05 | 3.90 |
| **Blood-loss anemia** | 0.01 | 0.01 | 10.94 | 0.01 | 0.01 | 13.15 | 0.01 | 0.01 | 10.94 |
| **Deficiency anemia** | 0.03 | 0.03 | 5.09 | 0.04 | 0.04 | 4.87 | 0.03 | 0.03 | 5.09 |
| **Alcohol abuse** | 0.04 | 0.04 | 4.52 | 0.06 | 0.06 | 3.61 | 0.04 | 0.04 | 4.52 |
| **Drug abuse** | 0.01 | 0.01 | 12.68 | 0.01 | 0.01 | 11.07 | 0.01 | 0.01 | 12.68 |
| **Psychoses** | 0.00 | 0.00 | 22.09 | 0.00 | 0.00 | 14.72 | 0.00 | 0.00 | 22.09 |
| **Depression** | 0.20 | 0.16 | 1.54 | 0.23 | 0.18 | 1.31 | 0.20 | 0.16 | 1.54 |

Table S9 Regression coefficients of Per Protocol analysis for health care use (balanced)

|  |  |  |  |
| --- | --- | --- | --- |
|  | DDD | Days in hospital | Sick-pay days |
|  |  |  |  |
| First year | 96.45*** | 1.20** | -1.28 |
|  | (25.25) | (0.51) | (2.10) |
| Second year | 213.16*** | 0.75 | -5.24** |
|  | (34.34) | (0.48) | (2.15) |
| Intervention | 0.00 | -0.00 | -0.00 |
|  | (75.98) | (0.43) | (2.64) |
| First year #intervention | 59.53 | -1.00 | -0.52 |
|  | (38.37) | (0.70) | (3.08) |
| Second year#intervention | 57.76 | -0.45 | 0.69 |
|  | (49.04) | (0.72) | (3.10) |
| TK (health insurance) | 22.07 | 0.57* | -0.76 |
|  | (86.14) | (0.33) | (1.88) |
| mhplus (health insurance) | 158.55 | -0.87 | -2.62 |
|  | (250.39) | (0.73) | (4.97) |
| Constant | 1,782.81*** | 2.64*** | 9.15*** |
|  | (79.20) | (0.36) | (2.14) |
|  |  |  |  |
| Observations | 3,388 | 3,388 | 3,388 |
| Patients | 1,370 | 1,370 | 1,370 |

Random-effects linear regression model: constant (cost of control group at baseline), intervention (difference from intervention group at baseline), first year (change in cost from baseline in control group), second year (change in cost from baseline in the control group), first year*intervention (DD estimator: cost difference after first year), second year*intervention (DD estimator: cost difference after second year), Robust standard errors in parentheses; ***p<0.01, **p<0.05, *p<0.1

Table S10 Regression coefficients of Per Protocol analysis for health care costs (balanced)

|  |  |  |  |  |  |  |  |  |  |  |
| --- | --- | --- | --- | --- | --- | --- | --- | --- | --- | --- |
|  | Total costs | Outpatient physician services | General practicioner | Outpatient non-physician services | Medical supplies | Hospital treatment | Sick pay | Medication | Rehabilitation | Prevention program |
|  |  |  |  |  |  |  |  |  |  |  |
| First year | 1,028.95** | 178.05 | 4.90*** | 12.69 | 34.57 | 685.12** | -17.97 | 89.20** | 42.00 | 0.16 |
|  | (425.55) | (138.86) | (1.81) | (12.50) | (27.29) | (321.19) | (140.50) | (43.56) | (26.15) | (0.61) |
| Second year | 1,190.12*** | 287.95* | 9.67*** | 35.68** | 158.58*** | 697.38** | -320.69** | 270.07*** | 59.54** | -0.66 |
|  | (459.34) | (158.72) | (2.91) | (15.33) | (38.23) | (341.60) | (140.36) | (87.16) | (23.70) | (0.98) |
| Intervention | -0.00 | 0.00 | 0.00 | 0.00 | 0.00 | -0.00 | -0.00 | 0.00 | 0.00 | 0.00 |
|  | (438.99) | (109.88) | (5.65) | (24.77) | (45.23) | (298.00) | (175.81) | (103.17) | (17.81) | (1.18) |
| First year #intervention | -595.50 | 159.73 | 4.62* | 9.02 | -17.97 | -699.57 | -27.45 | -16.44 | -34.07 | 0.36 |
|  | (589.42) | (203.26) | (2.80) | (16.85) | (44.26) | (441.48) | (216.68) | (62.03) | (33.28) | (1.22) |
| Second year#intervention | -226.07 | -36.55 | -6.97 | 20.02 | -52.45 | -549.73 | 118.91 | 319.04 | -29.13 | -0.34 |
|  | (738.57) | (182.89) | (4.34) | (22.38) | (55.61) | (488.85) | (210.59) | (325.30) | (34.19) | (1.47) |
| TK (health insurance) | 883.72** | 418.07*** | 10.44 | -56.21* | -33.30 | 457.64** | 5.34 | -8.13 | 43.42*** | -0.48 |
|  | (353.23) | (125.97) | (6.40) | (31.23) | (47.89) | (203.63) | (133.80) | (131.66) | (13.90) | (1.12) |
| mhplus (health insurance) | -325.56 | 82.20 | 31.77 | -156.83*** | -65.92 | -291.40 | -179.62 | 44.58 | -36.39*** | 0.39 |
|  | (838.05) | (145.74) | (24.88) | (33.26) | (70.78) | (553.88) | (276.77) | (213.92) | (9.65) | (3.10) |
| Constant | 4,390.12*** | 875.50*** | 21.26*** | 203.63*** | 228.16*** | 1,647.54*** | 511.51*** | 917.16*** | 21.77 | 3.03** |
|  | (334.61) | (86.43) | (5.34) | (29.60) | (43.89) | (216.04) | (142.64) | (114.16) | (13.50) | (1.19) |
|  |  |  |  |  |  |  |  |  |  |  |
| Observations | 3,388 | 3,388 | 3,388 | 3,388 | 3,388 | 3,388 | 3,388 | 3,388 | 3,388 | 3,388 |
| Patients | 1,370 | 1,370 | 1,370 | 1,370 | 1,370 | 1,370 | 1,370 | 1,370 | 1,370 | 1,370 |

Random-effects linear regression model: constant (cost of control group at baseline), intervention (difference from intervention group at baseline), first year (change in cost from baseline in control group), second year (change in cost from baseline in the control group), first year*intervention (DD estimator: cost difference after first year), second year*intervention (DD estimator: cost difference after second year), Robust standard errors in parentheses; ***p<0.01, **p<0.05, *p<0.1

Table S11 Regression coefficients of As Treated analysis for health care use (unbalanced)

|  |  |  |  |
| --- | --- | --- | --- |
|  | DDD | Days in hospital | Sick-pay days |
|  |  |  |  |
| First year | 118.02*** | 1.62*** | -2.81** |
|  | (22.16) | (0.46) | (1.40) |
| Second year | 250.41*** | 1.46** | -3.70** |
|  | (29.38) | (0.61) | (1.67) |
| Intervention | 2.18 | -0.16 | 2.21 |
|  | (66.30) | (0.36) | (2.27) |
| First year #intervention | 34.19 | -1.23* | 0.56 |
|  | (35.94) | (0.65) | (2.65) |
| Second year#intervention | 14.94 | -1.01 | -1.54 |
|  | (45.21) | (0.80) | (2.71) |
| TK (health insurance) | 19.71 | 0.09 | -0.82 |
|  | (68.28) | (0.39) | (1.39) |
| mhplus (health insurance) | -297.49* | -1.95*** | -0.32 |
|  | (166.11) | (0.55) | (3.88) |
| Constant | 1,807.31*** | 3.07*** | 7.88*** |
|  | (58.25) | (0.33) | (1.56) |
|  |  |  |  |
| Observations | 3,856 | 3,856 | 3,856 |
| Patients | 1,685 | 1,685 | 1,685 |

Random-effects linear regression model: constant (cost of control group at baseline), intervention (difference from intervention group at baseline), first year (change in cost from baseline in control group), second year (change in cost from baseline in the control group), first year*intervention (DD estimator: cost difference after first year), second year*intervention (DD estimator: cost difference after second year), Robust standard errors in parentheses; ***p<0.01, **p<0.05, *p<0.1

Table S12 Regression coefficients of As Treated analysis for health care costs (unbalanced)

|  |  |  |  |  |  |  |  |  |  |  |
| --- | --- | --- | --- | --- | --- | --- | --- | --- | --- | --- |
|  | Total costs | Outpatient physician services | General practicioner | Outpatient non-physician services | Medical supplies | Hospital treatment | Sick pay | Medication | Rehabilitation | Prevention program |
|  |  |  |  |  |  |  |  |  |  |  |
| First year | 1,269.25*** | 174.94 | 5.32** | 23.18** | 37.81 | 944.83*** | -109.50 | 187.19*** | 20.95 | 0.12 |
|  | (342.19) | (110.68) | (2.12) | (10.02) | (23.33) | (275.71) | (97.13) | (49.69) | (20.03) | (0.45) |
| Second year | 1,822.80*** | 303.85** | 7.10** | 49.99*** | 157.26*** | 1,018.95*** | -226.39** | 448.17*** | 55.13** | 0.51 |
|  | (439.38) | (130.07) | (2.88) | (13.74) | (36.59) | (335.93) | (93.81) | (132.86) | (22.69) | (0.84) |
| Intervention | 118.24 | 19.89 | -6.67 | 22.04 | 30.66 | -48.10 | 107.51 | 9.10 | -13.17 | 1.21 |
|  | (356.13) | (87.03) | (5.64) | (20.45) | (38.59) | (236.44) | (140.42) | (84.85) | (15.64) | (0.96) |
| First year #intervention | -630.87 | 152.73 | 3.48 | -0.97 | -31.57 | -766.46* | 102.26 | -112.44* | 5.53 | 0.15 |
|  | (530.33) | (185.76) | (3.01) | (15.18) | (41.03) | (397.13) | (197.09) | (66.05) | (29.81) | (1.13) |
| Second year#intervention | -773.56 | -56.69 | -4.92 | 4.15 | -50.31 | -758.64 | 23.00 | 133.90 | -22.21 | -1.70 |
|  | (707.85) | (156.99) | (4.25) | (21.14) | (55.78) | (470.97) | (175.67) | (332.21) | (32.53) | (1.33) |
| TK (health insurance) | 528.18 | 361.82*** | 10.62* | -42.63* | -35.30 | 206.31 | 20.98 | -65.10 | 29.99** | -0.52 |
|  | (322.01) | (98.71) | (5.69) | (22.93) | (35.40) | (223.00) | (92.05) | (109.77) | (14.50) | (0.74) |
| mhplus (health insurance) | -1,432.09** | -137.74 | 42.68* | -134.98*** | -36.09 | -1,031.13*** | -3.41 | -224.78 | -51.86*** | 1.76 |
|  | (605.51) | (86.17) | (21.89) | (26.01) | (59.54) | (357.27) | (222.41) | (154.45) | (11.41) | (2.48) |
| Constant | 4,492.29*** | 884.70*** | 29.74*** | 176.82*** | 214.54*** | 1,813.63*** | 412.08*** | 947.31*** | 40.26*** | 2.02*** |
|  | (258.90) | (53.07) | (4.97) | (18.86) | (31.82) | (183.48) | (92.10) | (90.18) | (13.45) | (0.57) |
|  |  |  |  |  |  |  |  |  |  |  |
| Observations | 3,856 | 3,856 | 3,856 | 3,856 | 3,856 | 3,856 | 3,856 | 3,856 | 3,856 | 3,856 |
| Patients | 1,685 | 1,685 | 1,685 | 1,685 | 1,685 | 1,685 | 1,685 | 1,685 | 1,685 | 1,685 |

Random-effects linear regression model: constant (cost of control group at baseline), intervention (difference from intervention group at baseline), first year (change in cost from baseline in control group), second year (change in cost from baseline in the control group), first year*intervention (DD estimator: cost difference after first year), second year*intervention (DD estimator: cost difference after second year), Robust standard errors in parentheses; ***p<0.01, **p<0.05, *p<0.1

Table S13 Entropy Balancing As Treated N=1685 (balanced for 1st moment, Interaction health insurance company)

|  | **Intervention** | | | **Control Pre Balancing** | | | **Control Post Balancing** | | |
| --- | --- | --- | --- | --- | --- | --- | --- | --- | --- |
|  | **Mean** | **Variance** | **Skewness** | **Mean** | **Variance** | **Skewness** | **Mean** | **Variance** | **Skewness** |
| **Sex (male)** | 0.72 | 0.20 | -1.00 | 0.68 | 0.22 | -0.75 | 0.72 | 0.20 | -1.00 |
| **Age** | 66.73 | 67.88 | -0.34 | 66.41 | 74.87 | -0.41 | 66.73 | 72.63 | -0.43 |
| **Status of health insurance: regularly insured** | 0.37 | 0.23 | 0.55 | 0.37 | 0.23 | 0.54 | 0.37 | 0.23 | 0.55 |
| **Status of health insurance: pensioner** | 0.62 | 0.24 | -0.50 | 0.60 | 0.24 | -0.41 | 0.62 | 0.24 | -0.50 |
| **Disease management program** | 0.39 | 0.24 | 0.46 | 0.37 | 0.23 | 0.55 | 0.39 | 0.24 | 0.46 |
| **Outpatient costs (€)** | 1085.05 | 883525.80 | 3.02 | 1096.02 | 847595.09 | 2.02 | 1085.05 | 830528.98 | 2.09 |
| **Outpatient non-physician services costs (€)** | 160.06 | 125175.96 | 4.18 | 142.97 | 142310.67 | 6.88 | 160.06 | 207064.14 | 6.46 |
| **Medical supplies costs (€)** | 203.22 | 677222.61 | 10.40 | 197.33 | 418382.91 | 6.95 | 203.22 | 446173.87 | 6.43 |
| **Hospital costs (€)** | 1931.87 | 25383136.88 | 6.68 | 1878.40 | 18712988.86 | 3.99 | 1931.87 | 20753472.21 | 4.06 |
| **Sick pay costs (€)** | 502.50 | 8387612.93 | 7.77 | 458.61 | 6204762.29 | 6.62 | 502.50 | 7137899.23 | 6.50 |
| **Medication costs (€)** | 913.77 | 2882949.92 | 6.66 | 905.94 | 3508424.82 | 8.01 | 913.77 | 3607075.85 | 7.78 |
| **Rehabilitation costs (€)** | 51.94 | 92848.08 | 6.25 | 55.72 | 120256.24 | 6.87 | 51.94 | 103249.17 | 6.87 |
| **Prevention training cost (€)** | 2.70 | 370.99 | 8.30 | 1.86 | 213.33 | 9.09 | 2.70 | 321.06 | 7.58 |
| **Total costs** | 4880.65 | 51738124.28 | 4.53 | 4776.27 | 37195278.93 | 2.86 | 4880.65 | 40282789.33 | 2.78 |
| **Health insurance company (TK)** | 0.71 | 0.21 | -0.94 | 0.64 | 0.23 | -0.58 | 0.71 | 0.21 | -0.94 |
| **Health insurance company (mhplus)** | 0.02 | 0.02 | 6.15 | 0.03 | 0.03 | 5.93 | 0.02 | 0.02 | 6.15 |
| **TK*Total costs** | 3570.91 | 47638209.38 | 5.13 | 3115.72 | 29738240.19 | 3.23 | 3570.91 | 35726446.08 | 3.13 |
| **mhplus*Total costs** | 127.14 | 1411864.43 | 13.81 | 74.51 | 632293.18 | 16.59 | 127.14 | 1398643.45 | 11.56 |
| **Daily defined dose** | 1801.34 | 1650072.55 | 1.20 | 1778.18 | 1600110.99 | 1.10 | 1801.34 | 1656358.31 | 1.06 |
| **TK*DDD** | 1290.31 | 1839628.24 | 1.28 | 1151.66 | 1731648.37 | 1.24 | 1290.31 | 1814070.47 | 1.10 |
| **mhplus*DDD** | 45.86 | 110551.96 | 8.28 | 35.16 | 70239.97 | 9.22 | 45.86 | 115674.38 | 8.47 |
| **Hospital days (number)** | 2.98 | 52.75 | 4.21 | 3.01 | 52.63 | 4.69 | 2.98 | 48.22 | 4.36 |
| **Sick pay days** | 8.38 | 1962.01 | 6.46 | 7.74 | 1653.24 | 6.35 | 8.38 | 1747.25 | 6.05 |
| **Congestive heart failure** | 0.13 | 0.12 | 2.17 | 0.13 | 0.11 | 2.25 | 0.13 | 0.12 | 2.17 |
| **Cardiac arrhythmia** | 0.19 | 0.15 | 1.62 | 0.19 | 0.15 | 1.61 | 0.19 | 0.15 | 1.62 |
| **Valvular disease** | 0.14 | 0.12 | 2.04 | 0.14 | 0.12 | 2.12 | 0.14 | 0.12 | 2.04 |
| **Pulmonary circulation disorder** | 0.03 | 0.03 | 5.66 | 0.03 | 0.02 | 6.08 | 0.03 | 0.03 | 5.66 |
| **Peripheral vascular disease** | 0.91 | 0.08 | -2.91 | 0.87 | 0.11 | -2.17 | 0.91 | 0.08 | -2.91 |
| **Hypertension, uncomplicated** | 0.78 | 0.17 | -1.33 | 0.81 | 0.15 | -1.58 | 0.78 | 0.17 | -1.33 |
| **Hypertension, complicated** | 0.16 | 0.13 | 1.86 | 0.16 | 0.13 | 1.90 | 0.16 | 0.13 | 1.86 |
| **Paralysis** | 0.01 | 0.01 | 8.19 | 0.02 | 0.02 | 6.77 | 0.01 | 0.01 | 8.19 |
| **Other neurological disorders** | 0.02 | 0.02 | 6.15 | 0.03 | 0.03 | 5.13 | 0.02 | 0.02 | 6.15 |
| **Chronic pulmonary disease** | 0.25 | 0.19 | 1.16 | 0.26 | 0.19 | 1.07 | 0.25 | 0.19 | 1.16 |
| **Diabetes, uncomplicated** | 0.30 | 0.21 | 0.85 | 0.29 | 0.21 | 0.94 | 0.30 | 0.21 | 0.85 |
| **Diabetes, complicated** | 0.19 | 0.16 | 1.57 | 0.23 | 0.17 | 1.31 | 0.19 | 0.16 | 1.57 |
| **Hypothyroidism** | 0.13 | 0.11 | 2.22 | 0.13 | 0.11 | 2.17 | 0.13 | 0.11 | 2.22 |
| **Renal failure** | 0.14 | 0.12 | 2.04 | 0.14 | 0.12 | 2.12 | 0.14 | 0.12 | 2.04 |
| **Liver disease** | 0.14 | 0.12 | 2.07 | 0.17 | 0.14 | 1.78 | 0.14 | 0.12 | 2.07 |
| **Peptic ulcer disease excluding bleeding** | 0.03 | 0.03 | 5.89 | 0.02 | 0.02 | 6.77 | 0.03 | 0.03 | 5.89 |
| **Lymphoma** | 0.00 | 0.00 | 15.56 | 0.01 | 0.01 | 11.07 | 0.00 | 0.00 | 15.56 |
| **Metastatic cancer** | 0.01 | 0.01 | 8.87 | 0.01 | 0.01 | 8.03 | 0.01 | 0.01 | 8.87 |
| **Solid tumor without metastasis** | 0.10 | 0.09 | 2.59 | 0.11 | 0.10 | 2.54 | 0.10 | 0.09 | 2.59 |
| **Rheumatoid arthritis/collagen vascular diseases** | 0.04 | 0.04 | 4.93 | 0.08 | 0.07 | 3.13 | 0.04 | 0.04 | 4.93 |
| **Coagulopathy** | 0.06 | 0.05 | 3.82 | 0.05 | 0.05 | 4.07 | 0.06 | 0.05 | 3.82 |
| **Obesity** | 0.21 | 0.17 | 1.41 | 0.23 | 0.18 | 1.31 | 0.21 | 0.17 | 1.41 |
| **Weight loss** | 0.01 | 0.01 | 12.66 | 0.02 | 0.02 | 6.23 | 0.01 | 0.01 | 12.66 |
| **Fluid and electrolyte disorders** | 0.06 | 0.05 | 3.90 | 0.04 | 0.04 | 4.86 | 0.06 | 0.05 | 3.90 |
| **Blood-loss anemia** | 0.01 | 0.01 | 10.93 | 0.01 | 0.01 | 13.14 | 0.01 | 0.01 | 10.93 |
| **Deficiency anemia** | 0.03 | 0.03 | 5.09 | 0.04 | 0.04 | 4.86 | 0.03 | 0.03 | 5.09 |
| **Alcohol abuse** | 0.04 | 0.04 | 4.51 | 0.06 | 0.06 | 3.61 | 0.04 | 0.04 | 4.51 |
| **Drug abuse** | 0.01 | 0.01 | 12.66 | 0.01 | 0.01 | 11.07 | 0.01 | 0.01 | 12.66 |
| **Psychoses** | 0.00 | 0.00 | 22.07 | 0.00 | 0.00 | 14.71 | 0.00 | 0.00 | 22.07 |
| **Depression** | 0.20 | 0.16 | 1.53 | 0.23 | 0.18 | 1.31 | 0.20 | 0.16 | 1.53 |

Table S14 Regression coefficients of As Treated analysis for health care use (balanced)

|  |  |  |  |
| --- | --- | --- | --- |
|  | DDD | Days in hospital | Sick-pay days |
|  |  |  |  |
| First year | 102.72*** | 1.49*** | -2.55 |
|  | (23.45) | (0.48) | (2.21) |
| Second year | 214.35*** | 1.21* | -5.94** |
|  | (31.68) | (0.63) | (2.33) |
| Intervention | 0.00 | -0.00 | -0.00 |
|  | (69.13) | (0.37) | (2.71) |
| First year #intervention | 49.84 | -1.11* | 0.42 |
|  | (36.74) | (0.66) | (3.14) |
| Second year#intervention | 51.39 | -0.76 | 0.87 |
|  | (46.73) | (0.81) | (3.14) |
| TK (health insurance) | -1.32 | 0.31 | -0.19 |
|  | (76.59) | (0.37) | (1.80) |
| mhplus (health insurance) | 70.77 | -0.79 | 0.43 |
|  | (214.90) | (0.73) | (5.37) |
| Constant | 1,813.35*** | 2.73*** | 9.64*** |
|  | (68.56) | (0.34) | (2.10) |
|  |  |  |  |
| Observations | 3,856 | 3,856 | 3,856 |
| Patients | 1,685 | 1,685 | 1,685 |

Random-effects linear regression model: constant (cost of control group at baseline), intervention (difference from intervention group at baseline), first year (change in cost from baseline in control group), second year (change in cost from baseline in the control group), first year*intervention (DD estimator: cost difference after first year), second year*intervention (DD estimator: cost difference after second year), Robust standard errors in parentheses; ***p<0.01, **p<0.05, *p<0.1

Table S15 Regression coefficients of As Treated analysis for health care use (balanced)

|  |  |  |  |  |  |  |  |  |  |  |
| --- | --- | --- | --- | --- | --- | --- | --- | --- | --- | --- |
|  | Total costs | Outpatient physician services | General practicioner | Outpatient non-physician services | Medical supplies | Hospital treatment | Sick pay | Medication | Rehabilitation | Prevention program |
|  |  |  |  |  |  |  |  |  |  |  |
| First year | 1,118.33*** | 177.04 | 4.22** | 10.61 | 29.39 | 848.55*** | -72.68 | 89.85** | 40.95* | -0.06 |
|  | (395.16) | (130.44) | (1.80) | (11.84) | (26.57) | (292.28) | (137.61) | (42.19) | (22.82) | (0.58) |
| Second year | 1,323.57*** | 277.52* | 8.40*** | 34.37** | 146.76*** | 869.81** | -331.96** | 256.16*** | 62.16*** | -0.09 |
|  | (468.94) | (152.07) | (2.95) | (14.53) | (36.99) | (354.93) | (138.32) | (75.45) | (21.88) | (1.04) |
| Intervention | -0.00 | 0.00 | 0.00 | 0.00 | 0.00 | -0.00 | -0.00 | 0.00 | -0.00 | 0.00 |
|  | (386.97) | (92.94) | (5.47) | (26.20) | (45.13) | (253.27) | (166.37) | (91.62) | (15.00) | (1.20) |
| First year #intervention | -484.54 | 150.72 | 4.58 | 12.27 | -20.62 | -671.22 | 65.21 | -16.38 | -14.28 | 0.32 |
|  | (566.81) | (197.71) | (2.79) | (16.43) | (42.77) | (409.35) | (219.66) | (60.53) | (31.73) | (1.18) |
| Second year#intervention | -277.02 | -27.80 | -6.22 | 20.07 | -36.06 | -605.66 | 129.38 | 326.86 | -29.13 | -1.16 |
|  | (726.73) | (176.10) | (4.30) | (21.63) | (55.51) | (484.73) | (202.61) | (313.72) | (31.97) | (1.48) |
| TK (health insurance) | 724.77** | 375.01*** | 13.48** | -46.77* | -50.97 | 308.76 | 52.02 | -23.91 | 25.94* | -0.71 |
|  | (332.01) | (110.57) | (5.68) | (28.20) | (46.93) | (207.24) | (120.83) | (114.84) | (14.87) | (1.09) |
| mhplus (health insurance) | 54.82 | 28.77 | 48.28* | -157.79*** | -63.91 | -81.48 | 30.43 | 29.59 | -46.33*** | 1.71 |
|  | (914.69) | (140.44) | (24.93) | (27.88) | (74.16) | (616.80) | (302.64) | (188.40) | (11.79) | (3.30) |
| Constant | 4,432.40*** | 890.71*** | 20.94*** | 202.37*** | 256.79*** | 1,667.66*** | 497.25*** | 920.74*** | 29.72** | 3.37*** |
|  | (297.89) | (73.54) | (4.79) | (25.79) | (45.32) | (193.68) | (127.12) | (96.05) | (12.72) | (1.05) |
|  |  |  |  |  |  |  |  |  |  |  |
| Observations | 3,856 | 3,856 | 3,856 | 3,856 | 3,856 | 3,856 | 3,856 | 3,856 | 3,856 | 3,856 |
| Patients | 1,685 | 1,685 | 1,685 | 1,685 | 1,685 | 1,685 | 1,685 | 1,685 | 1,685 | 1,685 |

Random-effects linear regression model: constant (cost of control group at baseline), intervention (difference from intervention group at baseline), first year (change in cost from baseline in control group), second year (change in cost from baseline in the control group), first year*intervention (DD estimator: cost difference after first year), second year*intervention (DD estimator: cost difference after second year), Robust standard errors in parentheses; ***p<0.01, **p<0.05, *p<0.1
